# Ethical thinking in occupational and environmental medicine: Commentaries from the Selikoff Fund for Occupational and Environmental Cancer Research

**DOI:** 10.1002/ajim.23328

**Published:** 2022-02-14

**Authors:** Sheldon W. Samuels, Knut Ringen, William N. Rom, Arthur Frank

**Affiliations:** ^1^ Executive Vice President Irving J. Selikoff Fund for Occupational and Environmental Cancer Research Alexandria Virginia USA; ^2^ Stoneturn Consultants; Senior Science Advisor The Center for Construction Research and Training Seattle Washington USA; ^3^ Department of Global and Environmental Health NYU School of Global Public Health New York New York USA; ^4^ Department of Occupational and Environmental Health Drexel Dornsife School of Public Health Philadelphia Pennsylvania USA

**Keywords:** ethics, global warming, medical student education, nuclear workers

## Abstract

A tribute to Dr. Irving J. Selikoff MD, the founder of this journal, is indeed welcome now more than two decades after his passing. He was known during his lifetime as the US Father of Environmental Medicine which at the time encompassed occupational medicine and much more as industry also polluted the general environment. The 1970s were a busy time as OSHA and the EPA were newly formed and high exposures to workers were no exception. Dr. Selikoff was a brave pioneer examining workers throughout the country and Canada, publicizing their exposures, and writing and presenting the scientific results. Industry was not always receptive and controlled an astounding amount of narrative, with the creation of the American Journal of Industrial Medicine filling a void of scientific need. We four authors write about the ethics of occupational health, the plight of nuclear energy workers, the climate crisis and opportunity for unions to engage workers, and the global march toward educating medical students on workers' health and safety. All four of us interacted with Dr. Selikoff during his tenure at Mount Sinai, and over the years joined each other in promoting his legacy. Toward that end we have written articles honoring his memory.

## Highlights

- Introduction
- Introduction Conversations with Irving's Ghost: The Struggle for More Light ‐ Dialogues with Dr. Selikoff Reconstructed.
- History of the Atomic Weapons Workers' Fight for Compensation: A Report from the Abyss of Occupational and Environmental Health.
- The Future of Nuclear Power.
- Global Warming: An Opportunity for Unions and Government to Embrace Renewable Energy Jobs.
- Worldwide Changes in Occupational Health Provision Over the Past Half Century.
- The Evolving Toolmaker: In dialectics of biology, history and ethics, humankind survives by changing tools of mind and matter. Dedicated to the memory of Irving J. Selikoff, MD.

## 1 INTRODUCTION

The Selikoff Fund for Occupational and Environmental Research stemmed from the Workplace Heath Fund of the Industrial Union Department (IUD), AFL‐CIO, under the leadership of IUD President Howard D. Samuel. It took its present form in 1992, in the last months of Irving Selikoff's life. He left what became the Fund's first donation in his will. Subsequent donations came from the asbestos insulation and auto workers unions, the families of asbestos disease victims, and government workers. With the death of Dr. Selikoff, Dr. Arthur Upton, a retired director of the National Cancer Institute, became the Fund's president, followed by Dr. William N. Rom of New York University, who had taken part of his training with Dr. Selikoff at Mt. Sinai. Selikoff Fund Secretary‐Treasurer is Dr. Arthur L. Frank, of Drexel University, who also took part of his training with Selikoff. The Fund's Executive Vice President Sheldon W. Samuels served pro bono in Dr. Selikoff's unit at Mt. Sinai and retired as Director of Health, Safety and Environment of the AFL‐CIO Industrial Union Department. Selikoff Fund board member Dr. Knut Ringen, principal partner of Stoneturn Consultants, served on the associate staff of the Department. Officers of the Selikoff Fund serve pro bono as Selikoff did.

The Fund's projects center on the special needs of nuclear weapons workers, from the mines to waste disposal. The needs for reduction of risks and protection of family‐supporting compensation for these workers are global.

**Tributes to Dr. Irving J. Selikoff:**

**Sheldon Samuels**

Sheldon W. Samuels, AB, Executive Vice President, Selikoff Fund for Occupational and Environmental Cancer Research. A former graduate student in the departments of philosophy and anthropology of The University of Chicago and a program director of the university's press, the Albert Schweitzer Education Foundation, NYS Health Department, USPHS, EPA and the Industrial Union Department, AFL‐CIO, he served on the faculty of Mount Sinai School of Medicine, Drexel School of Public Health and the State University of New York at Oneonta. He holds inter alia Bausch and Lomb, Rene Dubos, and Collegium Ramazzini awards. He served pro bono as Special Representative for Nuclear Weapons Workers of the Metal Trades Department, AFL‐CIO.

Dr. Irving J. Selikoff was a remarkable physician and scientist who contributed to knowledge in several areas of medicine, and who helped train a generation of occupational physicians who now work around the world. He spent most of his career at the Mount Sinai Hospital, and subsequently Mount Sinai's School of Medicine. He clearly left his mark at that institution, among many others.

Irving Selikoff attended medical school during World War II in Edinburgh, Scotland, interrupted by war activities which required one year of training in Australia. Completing his studies in Scotland, he returned to New York City where he began work at the Sea View Sanatorium on Staten Island. At that institution he focused on chest disease and was instrumental in the development of isoniazid, for many years a standard treatment for tuberculosis. His early scientific papers include research on amyloidosis and the efficacy of isoniazid on pregnant women. For this study he was honored with the Lasker Award in 1955.

With several colleagues he established a group practice in Patterson, New Jersey where he first began to see patients with asbestosis from the nearby Union Asbestos and Rubber Company (Unarco) asbestos plant. From this initial involvement he developed international studies looking at asbestos workers plus workers in a variety of crafts in North America and overseas. Dr. Selikoff published his findings on asbestos‐related disease widely. He was the first to point out the synergistic effect of carcinogens, such as those found in asbestos dust and cigarette smoke.

With the opening of the Mount Sinai School of Medicine in 1968, Dr. Selikoff established an occupational medicine residency program. A generation of occupational physicians trained by him work throughout the United States, Europe, and Israel. Mt. Sinai staff leader William Nicholson wrote the scientific case in the union petition for OSHA's first permanent standard, for the control of asbestos dust exposure. Dr. William Rom, as a resident, wrote the case for controlling lead exposure. A global perspective linking unions internationally was aided by resident Dr. Arthur Frank.

In the New York Academy of Sciences, Dr. Selikoff served as a Life Governor. He received numerous awards, honorary degrees, and honorary fellowships and served with distinction on the National Cancer Advisory Board. He founded, in 1980, the international Collegium Ramazzini and served for many years as its president. Dr. Selikoff fought studies conceived to support genetic modification as a substitute for environmental regulation. He supported ethically conceived studies in many fields of science. There is no mystery about why Selikoff was recognized internationally as a leading physician who significantly impacted the health of the whole of mankind.

**Knut Ringen**

I met Sheldon Samuels in 1978 when I was a study director at the US National Academy of Sciences and Samuels served on the expert committee for the project I was directing. At the end of the project, we created the Workers' Institute for Safety and Health (WISH) as a free‐standing technical organization within the labor movement, and a precursor to the Selikoff Fund. Samuels introduced me to Dr. Selikoff at Mount Sinai, who, together with Professors Ruth Lilis and Bill Nicholson, tutored me in occupational medicine.

Growing up in Norway I was introduced in high school to Henrik Ibsen's play *An Enemy of the People*, and its dramatic articulation of trade‐offs between health and economic priorities sparked my interest in public health. So, when I first heard Sheldon Samuels talk about necessary risks and what Irving Selikoff called the “Faustian Bargain,” I felt I was in a place where I was professionally engaged. This coincided in time with the criticality at the Three Mile Island nuclear power plant in Pennsylvania, and since then I have had a professional focus on balancing the huge risks and benefits of atomic power.

Selikoff and Samuels proposed a model for occupational high‐risk management which I implemented in a series of demonstration projects, including a project for former US Department of Energy Workers (BTMed. org) which is still in operation. This led to several assessments of safety in the nuclear industry, including one described in this issue. For the past 5 years I have led a team that provides independent oversight of safety and health within the Hanford Nuclear Weapons Reservation under an agreement established between the local trade unions and the employers.

**William N. Rom**

I was at the Environmental Sciences Laboratory with Dr. Selikoff from July 1, 1975 to June 30, 1977. I had written to him near the end of my internal medicine residency at the University of California, Davis/Sacramento Medical Center proposing 2 years' fellowship in Pulmonary and Occupational Medicine. Fortunately, my MPH from Harvard School of Public Health counted for a year in both fellowships so I could complete the program in two years. Dr. Alvin Teirstein accepted me into the pulmonary fellowship as well, and I obtained an American Lung Association/American Thoracic Society fellowship that covered most of my salary.

It was a whirlwind 2 years with Dr. Selikoff leading the charge to protect workers' health and discover new occupational diseases. We spent weekends in Paterson, New Jersey, examining asbestos workers, their wives, and children. Notably many had pleural plaques, lung fibrosis, and reduced lung function.^1^, ^2^ I found myself leading spirometry testing with Ray Warshaw, our able technician. Dr. Selikoff would always treat us to a kosher lunch! These humble beginnings taught us how to conduct environmental surveys across the country in union halls or public settings where we invited workers for research examinations: questionnaires, physical exam, blood draw, spirometry, and specialized tests. We pioneered the zinc protoporphyrin testing and nerve conduction studies in a lead‐acid battery plant in Indianapolis, fat biopsies in farmers in Michigan exposed to polybrominated biphenyls accidentally mixed into animal feed, sulfur dioxide exposure analysis in paper and pulp mills, styrene exposure, chloracne in polychlorinated biphenyl exposure in the Glens Falls General Electric plant, helium‐oxygen flow‐volume curves in garneting workers, and asbestos exposure in TVA power plants.^3^, ^4^, ^5^, ^6^ We spent two weeks in the Groton, CT, submarine rip‐out shipyard examining over 1000 workers exposed to asbestos. We noted for the first time that pleural plaques, especially among smokers, reduced lung function.^7^ I spent several weeks in the Immunology laboratories with Dr. J. Bekesi, and we published a Science paper showing reduced immune cells and their function in PBB‐exposed Michigan farmers.^8^ Dr. Selikoff gathered his team around him for almost daily readings of chest X‐rays according the ILO 12‐point scale.

At the beginning of my fellowship, I spent 3 weeks with Sheldon Samuels at the Industrial Union Department of the AFL‐CIO in Washington, D.C. Sheldon mentored me on union history, politics, environmental and worker protection, and the ethics and integrity of occupational medicine. He directed me to write a review of lead exposure in women and reproduction.^9^

Dr. Selikoff allowed me to spend half of my time in Pulmonary with Drs. Teirstein, Miller, and Siltzbach. Dr. Teirstein was a Master Clinician, and we entered into the era of computed tomography (CT scans). Dr. Siltzbach taught us about the Siltzbach‐Kveim Test and sarcoidosis. Dr. Miller reigned in the Pulmonary Function Laboratory where we observed unexpected longevity in a series of patients with severe kyphoscoliosis.^10^ I ended up passing both board exams in pulmonary and occupational medicine and was launched on an academic career.

Many years later I joined Dr. Selikoff in his New York Academy of Sciences conference on the third wave of asbestos‐related diseases. I spoke on the mechanisms of fibrosis in asbestos, silica, and coal‐exposed workers with alveolar macrophage growth factors stimulating fibroblast proliferation. After his retirement and after I had become the Pulmonary Division Chief at New York University Medical Center and Bellevue Chest Service Director, I visited him at Mount Sinai where he was still reading chest X‐rays with Dr. Kelly Rabin, and he advised me on becoming a leader in New York academic pulmonary medicine. Lastly, Dr. Selikoff had the important talent of recruiting outstanding faculty including Drs. Bill Nicholson, Ruth Lilis, Art Langer, Susan Daum, Henry Anderson, and many others.

**Arthur L. Frank**

Having had the privilege of joining the first class as Mount Sinai when it opened in 1968 as a medical school, I met Dr. Selikoff in December of that year when he invited the whole first‐year class of 36 to join him for lunch in his office. Many of us did, and over our “free” lunch, he shared with us much about his work with asbestos‐related disease among factory workers and un‐ionized insulators. Over the years, I was to learn he always had a large office and loved to entertain guests at mealtimes. I was struck by the coming together of many of my interests, cancer, the culture of working people, having been an anthropology major as an undergraduate, and the idea of field work. I approached him and asked for some one‐on‐one time.

At our personal meeting, he arranged for me to take my first elective time with a research colleague. Knowing of my potential interest in a career in academic medicine he offered to support me in adding PhD studies to my medical work, and also hired me to assist with his research. Now also enrolled in graduate school, over time, I graduated from doing paperwork to hands‐on physical examination of exposed workers.

I eventually became Mount Sinai's first MD/PhD student with Dr. Selikoff as my thesis chair. I had the opportunity to remain at the Mount Sinai Hospital for training in both internal medicine and with Dr. Selikoff and colleagues in occupational medicine with a two‐year break to serve as a commissioned officer in the U.S. Public Health Service in an NCI laboratory at the the NIH that Dr. Selikoff helped arrange.

After my training was completed, I joined the Mount Sinai faculty in his area and was put in charge of the residency program from which I had just graduated. I spent six years progressing in my academic career, the last two serving as the Scientific Administrator of his Environmental Services Laboratory, overseeing his budgets and personnel matters. This was good training for my next position as chair of a new department that I was hired to start—Preventive Medicine and Environmental Health—in the College of Medicine at the University of Kentucky.

I remained close to Dr. Selikoff until his death, and shortly before he died I was able to share with him that I had been asked to be guest editor of an issue of one of the two scientific journals he founded, and that we would honor his career and scientific contributions with papers from many admiring scientists from around the world.

Many lessons, both medical and otherwise, were learned by me and many others whom he worked with. Any history of occupational medicine in the United States would require acknowledgement of his many contributions.

## 2 CONVERSATIONS WITH IRVING'S GHOST: THE STRUGGLE FOR MORE LIGHT—DIALOGUES WITH DR. SELIKOFF RECONSTRUCTED

**Sheldon W. Samuels**

*“At the edge of a high bank formed by a ring of enormous broken rocks, we came to a halt and looked down on a crueler gathering. Choked by the overpowering assault of noxious stink that rose from the deep abyss, we drew back … ‘We must tarry here before we can go on,’ my master said, ‘till the sense has been resigned to the foul breath, and the odor will seem gone.’ ‘So the time will not seem wasted, can we find some compensation?’ I inquired of him, and he replied: That is what I have in mind.’”*

*“Wrapped around me was a cord … My leader ordered me to work it free … and taking it from me he flung it well beyond the precipice … and down it fell into the deep abyss.”*

*We explore to survive and learn to leave such hell! A gift at least borne of the changing human. “He spoke like a weary man who gasps for air: ‘Hold tight, we need such stairs to leave this place where there is so much evil everywhere.’”*

Dante Alighieri. *Inferno,* Cantos XI – XXXIV.

Early in April 1992, a Sunday morning, anchored in the *Fons Vitae*, my sloop, on a shallow of the western shore of the Chesapeake Bay, a few miles north of the confluence of the bay and the Potomac River. I heard the voice of a marine telephone operator: “Dr. Selikoff calling Mr. Samuels”.

The Friday before, I had visited my ill Mentor of Mentors to discuss the conclusion of nearly a quarter‐century of joint work. “Irving”, I responded into my radio, “why are you are calling?”

“It's the *Faustian Bargain*,” he replied softly.

“Repeat that”, I responded, “the connection is not clear!”

“It's the *Faustian Bargain*,” he repeated more loudly. “We need to emphasize the basic question of what should guide the work of science and medicine: *Faustian Bargains* or our *Moral Sense?”*

“It will help us understand,” I replied, “why radiation and other factors in the work environment persist in generating disease among nuclear weapons workers.”

“We need to explain why that question needs to be answered in all our work for every worker,” he said. “Cite what we have uncovered as examples of the persistent role of *Faustian Bargains* amid persistence of our moral sense in the pursuit of professional life.”

In my earlier dialogue with Selikoff, I found him on his hospital bed in great pain, yet trying to read Bertrand Russell's history of philosophy.

“Why that history?” I asked.

“It's short,” he said, “and I'm looking for some philosophic tradition that will help us!”

A “tradition that will help us!” The words of the son of a Talmudic scholar.

“That tradition,” I said, “is not in the history books. It exists, but is not taught. It would have to be reinstated.” And I described what I thought it was: Darwin's observations and beliefs interpreted, tempered, and expanded through the reconstructed minds of two predecessors, Baruch de Spinoza and Johann Wolfgang Goethe.

“Spinoza, Goethe, and Darwin?” he laughed. He laughed with greater force than I had heard from him in weeks. “Maybe. Maybe not. Maybe it's not so crazy. Will the scientists believe you? Who knows? Try it anyway.”

Selikoff's often repeated assertion: “It all began with Darwin,” incorporated the holistic social–biological integrated perspective of Darwin himself, not the tooth‐and‐claw version of the pseudo‐Darwinians. Selikoff would note that Darwin revolutionized medical research through his well‐founded authentication of forms of biological development in populations, concepts proven to be fruitful in the study of disease and associated social development in afflicted populations.

The views of early social scientists Adam Smith and Malthus helped cast the molds of “Darwin's population perspective,” as Selikoff would often say. Unfortunately, these views also encouraged distortions of Darwin's meaning. Thus, the expression “survival of the fittest” is not an expression used by Darwin, the social meaning of which is not consistent with his social views. Yet the concept has become an unacceptable rationalization of moral reality supposedly supported by Darwin.

An effect of this distortion is the acceptance of caste systems shaping the social structures of our species. In concurrent *ecumenes* of communication, workers and managers live in wholly or partially divided families, communities, cultures, religious congregations, recreational facilities, housing, peer groups (such as unions and industry associations), and political organizations. Separated educational and health care services funnel associated professionals and public servants into shared or concurrent castes. Resulting differences in social perspective make political conflict a norm.

Our journey, linking centuries of moral thinking on questions of science and technology in the realities of workers' lives, took a route mapped in the 18th century by Johann Wolfgang von Goethe's 12,111—line dramatic poem depicting the tragedy of a physician named *Faust*.^11^ Frustrated with learning and the persistent limits to his knowledge, power, and enjoyment of life, he chose a path in his career lit by the attention of the Devil, *Mephistopheles*. The Devil satisfies his quest for pleasure with lust for Gretchen, a virgin destroyed by deception and desire, a symbol of the human condition. *Faust* surrenders to the Devil through acts counter to the conduct of an ethical life.

The rejection of this behavior in the era of Goethe was seen in the America of Thomas Jefferson: belief in a moral instinct shared by humans. In our era Selikoff's abhorrence of *Faustian bargains* in the global development and implementation of occupational and environmental health laws exhibits the same instinct. The spirit is described by Goethe in his replication of the ancient miners' litany of the Harz Mountains and their eternal confrontations.^11^

In our own time, “*Mephistopheles* [remains a symbol of real life] … no mere evil principle, but a living person.” In the drama, *Mephistopheles* rescues *Faust* from fruitless study and brings him to a practiced life.^12^ In our real life, like *Faust*, we make pacts with the devil. “A solemn obligation,” not of a myth in another “world beyond,” is needed to counter the real devil serving us on earth as in *Faust*. For “unlimited pleasures” in a life in which—like *Faust*—we allow ourselves to be “cast in chains and perish in Hell.”^13^

One of Selikoff's favorite expressions in elaborating his belief in a moral instinct was: “It all began with Darwin!” Perusal of Darwin's work makes his point about an inherited alternative to the chains of *Faust*: a moral instinct, of “gestures which are innate or common to all individuals of the same species, … it is extremely doubtful, whether any of them were at first deliberately invented and consciously performed.” They were natural gestures.”^13^ What we witness is the ontogenetic and phylogenetic, clearly epigenetic reappearance of “primitive instincts” among lower species during human evolution, moral instincts.

There was never any doubt in Selikoff's mind about the underlying cause of ethical confrontation. "Man," Augustine observed, "can more easily count than be wise."^14^ Selikoff would have expressed this truth another way. “We lose sight of what is being counted. “Statistics,” he famously said, are “human beings with the tears wiped away.”

An effect of Augustine's underlying cause—the Bargain—can be found among those who are preoccupied with their own separate notions of what is good and what is true. The result that what ought to be found to be wise for us as a community eludes us. Selikoff would agree with Augustine that "there is wisdom: but whether there is one wisdom common to all or whether each wise man has his own as he has his own soul or mind, that we do not yet know."^14^

For Selikoff, this quandary of humankind is left not by wandering among the unknowable, but by engaging in another universe of discourse, the discourse of science. We engage with this universe of “as if,” by selecting a vision of the greatest good and the clearest truth framed by that which is most successful in preserving life, or as Augustine stated it, "the means by which every living thing flees death."

In his *Philosophie des Als Ob* (*Philosophy of As‐If*), Vaihinger argued that human beings can never really know the underlying reality of the world, and that as a result we construct systems of thought and then assume that these match reality.^15^ We behave "as if" the world matches our models. In particular, he used examples from the physical sciences, such as protons, electrons, and electromagnetic waves. None of these phenomena have been observed directly. Science posits that they exist, and uses observations made on these assumptions to create new and better constructs, i.e. they are heuristic [fruitful].

In Vaihinger, “the whole world of ideas is an instrument to enable us to orientate ourselves in the real world, but is a copy of that world … [they are] relatively objective ideational constructs [not] subjective or fictional ….”^15^

The difference between fiction and hypothesis is critical. For example when the Darwinian hypothesis is verified the fiction disappears, resulting in real explanation. Similarly, Goethe's schematic animal archetype (which I outline here) is a fiction justified as an expedient, according to Vaihinger, albeit Goethe himself saw it differently: dual possibilities within both the universes of “as if” and “as is”. “I soon felt the necessity of establishing a type,” the poet‐scientist wrote. From the perspective of “as is,” he described his search for real primordial starting points, “against which one might gauge all mammals for conformity and deviation; and just as I had once sought out the archetypal plant, I now sought to find the archetypal animal.”^16^ But in his search, he understood that if his findings were to be systematized and explained, not just an empirical, but a conceptual dimension is necessary.

Thus, Goethe claimed, “in an attempt to study the laws whereby life is given to organic nature … quite justifiably, a force was ascribed to this life for purposes of discourse; and this force could be, indeed had to be, assumed …. We [are] obliged to assume a double point of view, considering ourselves as an entity sometimes perceivable by the senses, and at other times recognizable only with the inner sense or noticed only by an effect.”^16^

Causation as a law of nature is just such an eternal idea. Variations bridge the worlds of “as if” and “as is.” Between these worlds is an abyss‐never‐filled, nor yet a void, which has expanded with the expansion of technology over the long eons of human evolution with mounting catastrophic strife in the struggle for life and freedom.

Clearly illustrated in the history of controlling atomic technologies, now centuries long, our failures multiply at an ever‐quickening pace from the difficulties of controlling our hands and their extensions—simple tools and complex technologies—with mind and reason. The living human being is not a preformed machine built, boxed and controlled by an isolated will for use at a random or self‐selected time. Each and together we are organic hierarchies of formative processes. Denoted from ancient times as “epigenesis,” each stage of these vital processes has come to be through prior causes. We become the set of prior causes of the next stage of organization.^17^ Formation is not explained by function, which explains usefulness. Use is not its cause or a mechanism, but a description of survival value for the individual and the population bearing the useful trait. Such is the case of our inherited moral instincts.

This abstract idea becomes a concrete dynamic that is more than a molecular biological dynamic. It is of course one form of a broader process recognized not only in biology, but in the social sciences as well since Aristotle, as a way of understanding a social analog. The genius of the oft‐quoted “experts” neglects an alternative to the *Faustian Bargain*: a moral sense! It has been long understood that human [and other than human] populations are never permanently without structure. Aristotle, writing centuries ago saw the village as one structure with inherited impulses among its inhabitants. Aristotle's Politics sets the moral tone: “But when several families are united, and the association aims at something more than the supply of daily needs, the first society to be formed is the village.”^18^

Within the village, “There are three things which make men good and virtuous; these are nature, habit, rational principle. In the first place, everyone must be born a man and not some other animal; so, too, he must have a certain character, both of body and soul.”^18^

An example of the moral sense in operation may be found in the modern history of nuclear technology and its community of scientists. Enrico Fermi worked toward bringing Heisenberg, Germany's leading atomic scientist, to America, “to prevent [Heisenberg] from working for Hitler,” [but Heisenberg feared that his emigration to the U.S.] “would bring something equally painful: pressure to work on a bomb intended for use against his homeland,” thus favoring one side of a two‐sided *Faustian Bargain*.^19^

“Explaining the “failure” of the German Bomb program by the “simple incompetence” or “lack of patriotism” of Heisenberg and other Germans “is not looking very hard.” Another mechanism was at work.

As horrendous as such issues may seem in an abstract sense, the plague of human ignorance on the virulence of the Faustian bargain coats depth and scope through millenia of human history and our future. In an abyss of ignorance, Faustian man alters the Face of the Earth, becoming a *slave of his own creation* in the struggle to maintain liberty in an environment dominated by money: cost–benefit analyses in which the sacrificial acceptance of death is seen.

“The last conflict is at hand,” Spengler warned, in which civilization is in its conclusive form: the conflict between money and blood.^20^ The private powers want free paths for their acquisition of greater resources. They want to make laws that use blood in the struggle for life itself, and with it the freedom to make the Devil's bargain, as seen in cost–benefit analyses, a process of changes in acceptable values in which, as Spengler notes, life seeks itself.^20^

To do “population thinking,” as Selikoff proposed, he understood that we must first consider the nature of living populations. They don't exist in a vacuum. They exist in an ecumene drawn and tied by communal mechanisms that facilitate organic actions—including changes in the ecumene and its members, such as modes of communication—enabling the life of the member and perpetuation of the member's species, such as modes of communication. The ecumenic environment as well as t‐he group environed is subject to cumulative change. *Ecumenes* can be insular, sheltered from external circumstances. In an ecumenic environment, “the system is the selecting agent.”^20^

Organization as an adaptive mechanism, Paul Weiss noted, in which “[t]he primacy of the organized state of a living system thus becomes axiomatic, and there is nothing in our practical experience in cellular and developmental biology that would justify the illusion that freely operating genes can be the “source” of organization of the developing system in the sense of imposing order de novo on an extra‐genetic matrix not already in possession of an organization of its own.”^21^ What does that mean?

In the last line of Albert Einstein's last published paper, a discussion of problems of incorporating causal theories of electromagnetic and gravitational fields in a unified system in physics—the theory of general relativity—he wrote that seeking a description of that reality is an attempt to find a purely algebraic theory.^22^ “But,” he wrote, “nobody knows how to obtain the basis for such a theory.”

The attempt to develop a general cosmology has a parallel in biological science: integrating theories of phylogeny or ontogeny reduced to physiological chemistry and molecular biology into holistic or organic models of living structures, both at the level of the nano and of the bifurcated mind and body, as done by the construction of epigenetic ecology, developing hierarchies or the suppositions of biological memory.

Some of the issues are intractable, such as found in the study of difficulties in applying population data to the causation of disease: the determination of cause of the same disease for a member of the population. Populations as subjects of study are difficult. The greatest mind of modern times clearly noted that reason is limited, not only his, but everybody's. Thus, Einstein answering fundamental questions of the creative human will said of some: *I do not know the answers, nor can anyone else know*.

Limits to reason alone as a path for answering our questions of what is good or true—even in the select dialectics of select gatherings of select scientists—is only one reality of the human will. Another is the failure to acknowledge the role of convention to accommodate the limitation of causal explanation, that is, the practical impossibility of tracing every causal pathway—leaving validation as the assumption of what is practically fruitful in answering questions of the meaning of observed fact, or useful contradictory explanations dependent on the heuristic acceptance of a convention.

Still another reality is willful distortion of methods or conclusions of investigation to fit individual or group needs or objectives, which themselves may be neither immoral nor false. The collective result is the plague of ignorance, endemic in human history, reflected in the inventive language of chance—“accidents”—used to explain what we observe as untraceably caused at any one time. “God doesn't play dice!,” says Einstein.^22^ Conventions can be used provided they are understood as conventions useful until such time as tracking problems are resolved.

Bernard Gert ventures that: “Rational persons want to avoid death, pain, disability, loss of freedom and loss of pleasure,” but will recognize differences among themselves on how to achieve these ends.^23^ Selikoff the scientist would negotiate these differences in the moral instinct at work by following the same kind of rules scientists follow as scientists: rules that are axioms, not given certainties, kept or dismissed by their fruitfulness or success in the work of preserving life.

The human condition is like a heaving Arctic ice pack. Constant friction between the floes and massive bergs close and open leads to sets of axioms from which we must choose in the pursuit of moral judgment. One channel with universal fruitfulness can be found by charting the preservation of human life measured not only by biological persistence of the species, but by the well‐being of the individual and the community, facets of life inseparable and no less valuable than the physiologic organism itself. Indeed, biologic persistence and the well‐being of the individual and of the community are not even knowable apart from each other. The flight from death is a flight from the death of all three of these.

The separation of biologic persistence, well‐being and community destroys the pathway between the three. The human is not a grain of sand, but by choice and by necessity a complex organism in an ecumene, a structured and directed environment that can be examined by unfettering the moral sense.

There is no *ecumene* in which the unity of the human multitude is not threatened by the conscious perpetuation of the ancient caste of workers and those who are associated with them: a form of aggression that imposes pestilence, deprivation, and the eugenic threat. Sociopaths embed the governing of the ecumene with economic sophistries. Cannibalism is justified in cost–benefit analyses used to compromise environmental justice, and shorten millions of lives. We have endured a century of workers compensation systems that unfairly transfer the burden of unnecessary uses of toxic agents and thus unnecessary sickness and death to the families of the afflicted, buried under distorted science and corrupted practices in medicine.

Castes, however, are not signified only by the color of one's collar: blue or white. Location, history, and economic or political status are other factors. Thus, we do not find it strange that “due to over four decades of uranium mining that supplied the US government and industry for nuclear weapons and energy, radiation illnesses characterize everyday Din'e (Navaho) life.”^24^

And this all takes place in full view for all who care to see, with not even a veil of shame. Franz Kafka, who earned his living in Prague in a workers' compensation bureau, understood what has been happening.

*“…Nobody can remain content with the mere knowledge of good and evil in itself, but must endeavor as well to act in accordance with it.” In this attempt, Kafka concludes, “[M]an is filled with fear; he prefers to annul his knowledge … yet the accomplished cannot be annulled, but only confused. It was for this purpose that our rationalizations were created. The whole world is full of them; indeed, the whole visible world is perhaps nothing more than the rationalizations of a man who wants to find peace of a moment.”* ^25^

Yet when it is evil that is being rationalized at the cost of life, we cannot accept even the peace of a moment: every living thing flees death first by demanding that the rationalization of the evil is known and understood, not only by the victim but throughout the ecumene.

Selikoff the optimist disliked my frequent use of the term “cannibalism” to describe the rationalization of evil in the work environment; he believed that it imprints too many negative images, slowing the process of positive dialogue (although he never argued that cannibalism was not alive and well in our space and time). Instead, he sought another framework of explanation that could lead to a fruitful substitute in achieving the primary human objective: the preservation of life.

Irving J. Selikoff, MD was a very private person. He did not want the usual biography focused on his personal life. He was far from shy and he sought recognition, but he had priorities. Conspicuous immortality was not his highest objective. He knew that the charisma of a leader is an important vector in the achievement of any agenda; there is no technician's black box for guiding human progress. It is appropriate to uncover a sense of the man or woman, but the critical idea that becomes an ideal towards which society must move is more important.

Soon after he retired in 1985, Selikoff began examining the moral content of the developing systems of beliefs and practices he and other physicians and scientists employ in discovering and managing the risks of workers to environmental disease. He was convinced that these beliefs and practices should be judged by their fruitfulness in the preservation of life, including the traditions that shape them, and that we ought to illuminate the forces of selection in their evolution. His message was aimed at his colleagues in medicine and science, but he wanted to reach his followers in government, the labor movement, and even some in industry as well.

He began to link ideas and their origin with people and events in his professional life that cast light on the agenda to which he was dedicated, indeed, with which he was preoccupied to his last hours.^26^

The project was not an exercise of warm memories or interesting speculation. The foremost challenge of occupational and environmental health then and now has been in the design of studies in the laboratory and in the clinic, even routine medical surveillance, and in the critical interpretation of the resulting information. The significant dialogue on design and interpretation is not taking place in the seminar or the academic conference, or even published in most of our learned journals. It has been taking place in the courtroom, the legislative hearing, and behind closed doors in the regulatory agencies. The resulting dialectic, not understood by the public nor fully appreciated by the “experts,” is a competition of systems of beliefs and practices each of which select different paths in the fate and well‐being of hundreds of millions of humans now and in the future.

At the very least, Selikoff not only wanted to understand his own system, but he also wanted to examine the beliefs and practices of those whom he both supported and contested on issues of science or public policy. Most importantly, he wanted to promote a broader understanding among them of the choices implicit in their systems and, if they prevail, before the people. He wanted to drain a swamp of mistakes.

Selikoff wanted to understand how key scientific concepts, including their moral history, are linked collectively to events in our lifetimes and in the lives of those before us that seem to persist and repeat, sometimes on the same and sometimes on different strands of time and space. This was to be an examination of tradition in its fullest dimensions—the logic and politics of its community at the base of discovery, and how it was all held together by the core intuition of the intuitive scientist: unification of what we know in the singular goal of the simplest, successful explanation. The moral nature of traditions of discovery and explanation found in the clinic and laboratory, he appreciated, may be discovered in the arts and literature of the past and present. He had in mind the creations of those who, like Goethe, are both scientists and poets or philosophers. Indeed, in that first discussion, he spoke of the virtue of Goethe's *Faust* seeking, but failing to attempt without corruption, to “perceive the inmost force that bonds the very universe.”^11^

Goethe depicts surrendering to the devil reminiscent of both the ethical life seen in Jefferson's belief in a moral instinct in the same era, and of Selikoff's abhorrence of the *Faustian Bargain* as seen in the governance of professional life in the United States and the western community, a condition encountered in implementation of the work health laws.

Perceptions that persist or are repeated, whether of scientists or common men, are at the base of such traditions. Perceptions are also judgments. What we see is often what we want to end, often the tails of problems we have not solved, obstacles we have not overcome, and tragedies we ought not tolerate.

Selikoff was in many ways a Jeffersonian who would more often than not assign collective responsibility for the moral mistakes we make, and not ascribe personal blame to the individual. He saw great difficulty in judging the extent to which an individual is fully a moral agent in the usually partially‐understood circumstances of most cases. This is an important nuance in understanding his optimistic behavior toward those on the other side of the struggles in which he was engaged.

Irving Selikoff's philosophy of optimism, his “opaque glass half full,” is a “perspective” (to use one of his favorite words) of the system of natural science and moral belief of a giant he idolized: Darwin. Both dissented from the establishment scientists of their day. Darwin, in addition, was seen as a heretic by the leaders of his community because of views that questioned articles of faith and thus, for some, of morality, views in a tradition of dissent at least as ancient in the British Isles as that of the 5th century Celtic monk Pelagius. Like Darwin, he was educated in theology, but was never ordained. They both believed, as did Selikoff and those in his tradition, in the ability of humankind through science to alter the environment to which we as organisms adapt. To quote John Dewey, we need “to expand and enrich experience” for “self‐creation and self‐regulation,” limited morally and intellectually only by the “defects in our good will and knowledge.”^27^

If we look for uniformity in this moral perspective among those who also held to the biology of Darwin, we will be disappointed. Dewey, a dominating optimist of Selikoff's strand of time and space, had learned and accepted Darwinian evolution from a work of T.H. Huxley, but had faith in human transformation through education, rejecting an inherent immutable evil in humankind. Huxley, Darwin's stalwart supporter and publicist, accepted most of Darwin's biology, but also believed that “the Eden would have his serpent.”^28^ Yet the divergence in beliefs did not necessitate a difference in moral judgment. At the exit of the complex of paths that can be taken by the developing moral sense, one finds a Dewey the optimist and a Huxley the pessimist both abhorring eugenics and other injustices rationalized by abused science.

Without question, events occur in our lives that shake the optimism even of a Darwin, Dewey or Selikoff. The opaque glass often appears to be half empty. Moral sense does not always penetrate the human surface. What Selikoff would call a moral mistake is frequently repeated, repetition that appears to be the constant reality, so that some may conclude that they are not moral mistakes. They are biologically‐evolved inherent evil.

Arthur Koestler, a leading pessimist of Selikoff's generation, posed “the possibility that Homo sapiens is a victim of one of evolution's countless mistakes,” of faulty brain design.^29^ Our species, he emphasized, “is virtually unique in the animal kingdom in (our) lack of instinctive safeguards against the killing of … members of (our) own species. … Man is alone … in practicing intraspecific murder on an individual and collective scale.” He pointed to the “striking disparity … between the growth‐curves of science and technology on the one hand and of ethical conduct on the other.”^29^

*Evidence that Koestler might be right is found among the banalities of diseases of work, where one need look no further on the periodic table than the misuse of atomic materials, such as beryllium. The unnecessary persistence of disease with which beryllium is associated adds credence to Koestler's hypothesis and the claims of the philosophy of pessimism*.

Selikoff sought his strain of optimism in the world of as if we inhabit. Our subjective predisposition to one strain or another, he believed, colors the whole of our *Weltanschauung*. Selikoff was an optimist in the same sense as was Goethe. They saw the wine glass of life as half full, not half empty. Far from vetting failure, it supported human hope. It was a universality guided by an idea of freedom and an ancient vision:

*“Every valley shall be exalted, and every mountain and hill shall be made low: and the crooked shall be made straight and the rough places plain …” (Isaiah 40:4)*

## 3 HISTORY OF THE ATOMIC WEAPONS WORKERS' FIGHT FOR COMPENSATION: A REPORT FROM THE ABYSS OF OCCUPATIONAL AND ENVIRONMENTAL HEALTH

**Sheldon W. Samuels**

**The Burden of Trinity**

At 5:30 in the morning of July 16, 1945, the loudspeakers emitting surreal strains of Tchaikovsky's “Serenade for Strings,” the first atom bomb was tested. Enrico Fermi estimated that the explosion was equivalent to the blast of 20,000 tons of TNT. The site was “Trinity”: 35 miles east of the Rio Grande, on a flat just west of the slopes of the Sierra Oscura, 18 miles south of Bingham, New Mexico, and 300 miles upwind from the city of Amarillo, Texas. The first bomb cost two billion 1945 dollars.^30^ The shock waves have not abated. What originated as a game in the scientists' world of “as if” has become a challenge to human existence.

The first warnings were found in diseases of nuclear workers, the sorrows of their families and social sickness in their communities, as witnessed by Laura Fermi in Chicago, in 1957. There she wrote *Atoms in the Family* and *Atoms for the World*.^31^, ^32^ The last was her account of the first Geneva conference on the peaceful uses of nuclear energy.

We learn that early in the history of the nuclear industry, in 1940, her husband Enrico Fermi's research assistant became fatally ill of beryllium disease from exposure to fumes in an unvented laboratory at Columbia University.^31^ Despite this case and European reports, the Public Health Service had in 1943 declared beryllium dust to be nontoxic.^33^ A few years later, Harriett Hardy, then medical officer at Los Alamos National Laboratory, encountered more cases of beryllium disease.^34^

Laura Fermi had lived in Los Alamos and worked in the LANL medical office. She was aware of the growing burden of occupational disease associated with the unleashing of nuclear energy. Years later, as historian for the Geneva policymaking conference in 1955, she remarked, fatefully, that “the dangers were cloaked in the mission of science in war.”

The nuclear industry her husband Enrico Fermi and his colleagues initiated began in 1952 with the Metallurgical Laboratory at The University of Chicago. Primarily a pile of uranium bricks in the squash court under the stands of the football stadium, it was later replaced by Argonne‐East and the Fermi National Accelerator Laboratories. Among those who worked in those laboratories, 668 were victims of cancer. Professor Fermi also died of cancer, but a link to his occupational exposures was not made. The federal government began to pay limited compensation and medical benefits.

High risks of radiation disease had been demonstrated and published in the prior century, at least since 1896, among workmen in Roentgen's laboratory.^31^ The recording of disease among miners in the *Erzgebirge,* a mountain region of Saxony, began even earlier. After 1942, small‐scale operations in laboratories, mining, ore processing, and fabrication plants, were replaced by a massive industry. While better understood today, the full spectrum of human health and ecological risks associated with radiation and other toxic agents in the atomic energy industry has yet to be fully recognized and faced. Professional and industrial workers still function with health, social and economic protections shaped by the traditional—sometimes primitive—practices of small academic laboratories.

Fears Laura Fermi expressed were based on observations in the research communities of Milan, New York, Los Alamos, and Chicago where she and Enrico had lived and worked. The biggest problems, she noted, result from reprocessing spent nuclear fuel containing long‐lived nuclear fission products such as Cesium 137 and Strontium 90. The nature of the early nuclear weapons program is seen in how a great scientist—Seaborg—stored his first fraction of plutonium: pasted to a cardboard strip in an old cigar box.^35^ Writing on the use of the new technology in power production, she noted that “physicists and the engineers … belittled the dangers to populations … they … did not deny the potential hazards. … [The risks were] “accepted as unavoidable.”^32^

There are now tens of thousands of nuclear weapons and thousands of tons of nuclear weapons materials in nine countries, the cost of which—including social costs—have not been reliably calculated. Aside from medical and other industrial applications, there also is a developing nuclear power industry, which provides 14% of the world's commercial electricity. The industry's 439 reactors produce 372 gigawatts in 31 countries and 35 more reactors under construction, nine in the United States. The American industry alone supports 400 nuclear suppliers. Roughly half the fuel for the American power reactors is blended‐down Russian bomb‐grade uranium. Enrichment plants are being built.^36^ Led by China, India, and Russia, *Reuters News* reports, more than 100 new reactors will be built over the next decade to reduce dependence on greenhouse gas‐producing power sources such as coal. The demand for new uranium mining, currently a small industry dominated by Canada, is expected to rise and new mines and mills are expected to open in Russia, Kazakhstan, and Australia.

Social costs measured by occupational disease among miners, millers, transporters, fabricators, weapons assemblers, power station operators, fuel recyclers, and waste handlers of uranium and other minerals and chemicals essential to nuclear energy generation have been largely hidden. Unmeasured financial costs have shifted to the families and communities, part of a legacy of ignorance and willful underestimation of the burden of occupational disease.

Early studies of these workers focused attention on cancer risks of miners exposed to high levels of radiation and other carcinogens. The cardinal Public Health Service study by Victor Archer, J.D. Gillam, and Joe Wagoner^37^ found more. Arizona, Colorado, Nevada, New Mexico, and Utah were experiencing the highest rates of suicide in America. This pattern of excess death has marked social sickness associated with uranium mining since its beginnings in the *Erzgebirge* of Central Europe centuries ago.

**Moral History**

Human ecological factors—the caste (a human group within a population perceived to be better or worse than others) and the *ecumene* (the region where members of castes live) are often inadequately considered in programs of disease prevention, intervention, and therapy, in which health beliefs and behaviors, and, ultimately, health status are shaped. Caste‐bifurcated communications networks influence health‐promoting actions, affecting the perceived need for services and the individual or group decision to seek medical assistance. Our attention is diverted by economic encounters between the physician and patient. Polarized by communication through the bifurcated social networks of the community, especially family and peer groups, the milieu in which we try to promote screening, monitoring, and early detection of disease is skewed. Yet, we often act *as if* the caste structure and its meaning for disease does not exist

The experience of this writer is that American workers and their families at the Department of Energy weapons facilities perceive the risks of work and the intent of programs to manage them. Their perceptions are refracted through participation in their union committees and environmental groups. Their vision is shaped by mass media and by what they are told by their employer. They are generally aware of, but “see” differently, recognized etiologic factors of disease in their work environment, including asbestos, ionizing radiation, beryllium dust, and other agents. Union and other leaders in the workers' caste “see” higher risks of disease among those inside the caste of workers than those outside the caste. Beyond general awareness of publicized chemical and physical agents of recognized diseases, they “see” another set of agents difficult to assess because of obscuring psychological, social, and moral despair within their caste. Suicide is the traditional, measurable effect.

Observing these phenomena a century ago, Emil Durkheim insisted that “…*one can ascertain…that life itself loses its attractions, that evil increases, or the causes of suffering increase, or the resistive force of individuals is reduced. If, then, we possess an objective and measurable fact translating the variations of intensity through which this sentiment passes in societies, we shall be able with one stroke to measure those of the average unhappiness in these same environments. This fact is the number of suicides.”* ^38^

This fact is more than an isolate either of economic deprivation or elevated status. As history teaches us, the established primary factors are recognized in the disorganization of the social organism, as reflected in impacts on the individual of political and cultural despair within the ecumene and in the caste. These changes are marked by rising rates of suicide: the consequence of social disorder and disintegration.

A critical ecological dynamic begins with changes in employer–employee relations. During such times the need for unifying symbols in society and the need to recognize collective moral principles are critical. The workers' sense of social unity is diminished when they are disabled or sick and unable to participate in normal work and community activities. Facilitating mechanisms—such as support groups—for handling these realities of the workplace are the responsibility of those able to respond to these needs. Acting on that responsibility is more likely to occur among those who share a community with the afflicted.

Solidarity alone is insufficient. Trusted information must be brought to the group. But looking outside the group for sources, the worker often sees a barren landscape. It is not unusual to find the absence of independent sources of trusted medical counsel among a community's medical providers. This factor points to the importance of structuring interventions to reinforce access to independent medical judgment that could help interpret information on occupational disease, psychosocial stress, and genetic or other tests. *The worker or the worker's family typically manage this cascade of issues without assistance, making determinations with little guidance, on problems that defy expert judgment, in the separation of occupational and nonoccupational causes of disease, modes of treatment, and options for economic relief within the systems of insurance and healthcare*. They typically lack effective networks and support structures, without which workers' ability to perform critical social and economic roles may be reduced to the point of nonexistence.

**The Legacy of the** ***Erzgebirge***

Predating the American uranium experience, in the *Erzgebirge* (Ore Mountains) of Germany and the Czech Republic, we encounter the latent, endemic, sentinel of a tradition of malignancy identified by Tomas Masaryk before he became the first president of Czechoslovakia. In Bohemia (among the Saxon miners of Joachimstal, now called Jachymov) uranium disease and community pollution were described centuries before by Agricola, town physician there and in Chemnitz (the capital of the region) in *De Re Metallica*: “… Miners are sometimes killed by pestilential air which they breathe; sometimes their lungs rot away.”^39^ On the north slopes, Jachymov lung disease was called Schneeberg lung disease, its name taken from a legendary mining town in Saxony, and established as lung cancer by German scientists Harting and Hesse in 1879.^40^

The history of the region supports Masaryk's account of suicide as a social malignancy among miners and their communities, measuring the despair associated with the impact of mining conditions, not only on the miners, but on the families and communities of miners over long periods of time. He read Goethe's accounts of conditions in the mining industry of the region, depicting centuries of disease, ecological devastation, and communal resignation—*entsagung*—pervading the life of the encapsulated mining communities of Saxony. As Minister of Mines, Goethe supervised the miners of Ilmenau.^41^ The resignation he observed, un‐bottled, becomes the suicide ritual of the region. His fiction imitates reality, echoed in the statistical analysis that enabled Masaryk a century later to predict expanding pools of human despair within the caste of workers, within which suicide takes place in widening dimensions. The suicide rates in Europe were highest on the northern slopes of the mountains, where the mines are concentrated, “the great increase in suicide among children” as well as adults, explained in part by occupation.

This writer's visits in the region confirmed a fact of moral history, a marker of the caste system: culturally‐influenced evasion of responsibility for unnecessary occupational disease. This legacy of the *Erzgebirge* is reflected in the fact that Schneeberg lung disease, diagnosed as lung cancer in 1879, is “commonly ascribed to a secondary effect of silicosis in an inbred population predisposed by hereditary susceptibility.”^42^ Denominating disease as a peculiarity of a region or of a people means one thing to scientists entrenched in traditions of biological causality ascribed to multiple risk factors, and means quite another to those with an economic or ideological interest in escaping responsibility for preventing, treating, and compensating occupational disease.

The genetic factors for this disease in these ancient communities have been aggressively explored by Dr. Hans Woitowitz and his colleagues. During the Cold War, Wismut, the uranium mining company in East Germany, employed 500,000–600,000 people, many prisoners of war or politics. Of about 9000 reported cases of lung cancer, about 5300 were compensated as occupational disease. Among these workers, Dr. Woitowitz' team of scientists found DNA damage in cells of former Wismut workers who have lung cancer. Their studies of the increase in unrepaired DNA “clarify the clinical relevance of genetic susceptibility to lung cancer with regard to the role of DNA damage and repair.” The investigators established that “a major role of six [cancer risk predisposing] ATM gene mutations could not be revealed for cancer predisposition.”^43^

Navajo miners of uranium, prisoners of abject poverty and caste discrimination, many of whom (like their German counterparts) lived in homes made of radioactive mine tailings. Successful Navajo claims have been awarded for a limited list of compensable occupational diseases, including lung cancer. [M Merritt, MD, personal communication] In America, this study was begun by Victor Archer and his colleagues.^37^ They discovered a pattern of excess cancer deaths associated with suicide among uranium miners in the Four Corners region—Arizona, Colorado, New Mexico, and Utah—a region experiencing four of the five highest rates of suicide in the United States. One of the earliest mines—Uravan—was opened in 1881. Archer et al. illustrated the effect of cultural differences on suicide rates predicted by Durkheim and Masaryk: between “white” and “American Indian” uranium miners. This classic work compared suicide rates in the male nonwhite population of Arizona and New Mexico to that of Indian miners, and the male white population of the United States as referent for white miners. Among 107 Indian miners, 9.7 deaths were expected due to suicide, but only five were observed. Among 745 white miners, 17.7 deaths due to suicide were expected, but 22 were observed.

That environment causes traditional occupational disease in these mines—dust and radon—never have been seriously disputed. Human tragedy had been unfolding in the uranium mines and mills, and was documented from the 1950s by Duncan Holaday and his handful of colleagues in the agency now known as the National Institute for Occupational Safety and Health, aided by doctors of the Indian Health Service.^44^ Based on their work, then‐Secretary of Labor Willard Wirtz testified in 1967 that endemic silicosis and cancer could have been prevented by enforcement of a 1936 federal mine safety law. The adopted radon standard still in force in the mines and mills is less stringent than Secretary Wirtz proposed, and four times greater than a feasible standard recommended by NIOSH.

Environmental factors in occupational suicide, while long noted and diverse, are not usually identified or accepted. Yet an early study by Mancuso and Locke found an excess of suicide in viscose rayon workers in which exposure to carbon disulfide was the suspected cause.^45^ A later review of the literature by Boxer, Burnett, and Swanson disputed this and similar findings, concluding that “any causal relationship between suicide and job‐related factors” was not proven.^46^ The authors attributed the deaths to personal factors, such as alcoholism. However, Kposowa studied suicides among workers in construction and manufacturing, and identified associations with neurotoxic substances, as well as other stressors, including threats to job security, malpractice lawsuits, and various forms of harassment.^47^ Their work is strengthened by workers' experience in vinyl chloride manufacture. On the day the new OSHA standard for this material went into effect, a tire worker in Western Maryland “flunked” the bilirubin test required by the standard and shot himself. At a vinyl chloride plant in Louisville, Kentucky, “failed” workers were put to work in a pallet plant, which they quickly named the “leper colony.”

NIOSH was excluded from the DOE system except for the uranium & beryllium mines and mills, so that their knowledge of what went on in the laboratories and plants was restricted. In the uranium and beryllium operations, from the 50s, NIOSH initiated critical studies that uncovered hazardous conditions and established fact‐based criteria for environmental and medical monitoring standards. Union efforts resulting in the existing radon standard covering the mines and mills were based on the work of NIOSH personnel, saving hundreds of lives.

To understand the full burden of disease, studies of the effects of low doses of ionizing radiation and other agents in the work environment on non‐cancer mortality and morbidity are needed. Much more needs to be known before the full toll can be understood, unnecessary risk prevented, and the costs of past and residual risk fairly distributed. Nevertheless, decades since Trinity, the effects of some aftershocks remain in the workers' compensation programs for nuclear industry workers.

The issues are not unique to this industry. They plague the entire caste of workers. Only a small fraction of the burden of occupational disease is ever compensated. Consistent with the quintessential character of life in the caste, the social and economic costs of occupational disease and injury are borne primarily by the worker and the worker's family, directly in wage loss and uncompensated healthcare costs not covered by insurance, and indirectly through increased insurance premiums and payroll taxes that finance social security disability and medical retirement benefits.^48^ The immense size of the problem, and the inability of our compensation systems to deal equitably with the issues, was made startlingly clear in the coal miners' struggle to achieve passage of the Black Lung Benefits Reform Act of 1977.

In the past, the Amarillo metropolitan area, home to the Pantex nuclear weapons assembly plant, was historically afflicted with significant point sources of particulates and sulfur oxides. The particulates of concern were asbestos and metals, especially beryllium. The agent of greatest concern, however, was ionizing radiation. High levels of anxiety were generated by a lack of environmental (area and personal) and medical monitoring and the absence of personal exposure records. Management and governmental assurances of no‐effect exposures were companions to slack controls and minimal safe‐practice training. Liaison with the local medical community and integration of known environmental factors into health care and wellness programs did not exist. Even if a family physician was curious about workplace exposures, plant management seldom transmitted information of value to the examining provider. Thus, personal medical records were incomplete.

Subcontractor, temporary, probationary, and short‐term employees who when exposed to known high levels of radiation were, in the words of one participant, “flushed.” They are not fully represented in the records or in any study of this and sister populations. In any one cell of high exposure, there typically might have been only one permanent employee.

It is important to keep in mind that in the early days of nuclear weapons production, fabrication at Pantex was conducted under wartime conditions that have been moderated, but not eliminated, over time. To some extent, this explains cavalier work procedures, personnel policies insensitive to levels or duration of exposure, and unacceptable materials handling.

In meetings convened by the Metal Trades Department of the AFL‐CIO in Amarillo of councils of nuclear workers from throughout the DOE system, perceptions of the workers focused on cases of disease reasonably attributable to the work environment. High levels of anxiety and anger had overcome the natural reticence of the patriot‐worker to complain. Peer pressures had changed, and the stoic hesitancy to express personal pain endemically characteristic of their culture was pierced. Information on conditions and standard practices of the past and present became more specific and expressed more strongly. The high concern over the absence of adequate personal medical and exposure records was a major factor in the call for energy workers' compensation legislation.

The follow‐up mechanism was the MTD‐IUD Workplace Health Fund (WHF) of the AFL‐CIO. With the dismantling of the Industrial Union Department of the AFL‐CIO in 1996, the program was taken over by *The Selikoff Fund for Environmental and Occupational Cancer Research*.

The Fund's first project was a sociological probe among Oak Ridge workers to guide the design of local work environment programs. The result of these efforts clarified the demand for providers independent of the usual DOE contractors and affiliates. Equally important, local programs were initiated to support the role of the local unions and their councils in the prevention, medical surveillance, and compensation of disease.

The second project was cancer research among former and current nuclear weapons workers and their families in the Department of Energy's Pantex facility in Amarillo by Dr. William Rom. Supported by the National Cancer Institute, he explored the use of sputum cytology in lung cancer screening, similar to work that had been initiated in Grand Junction for uranium miners. The research resulted in rejection of those methods of identifying diseased workers.

A third project conducted by Dr. Arthur Frank is a clinic established in Amarillo, using independent Texas providers he trains and supervises in continuous surveillance of nuclear weapons workers of the Pantex facility and their families.

**An End to Slow Progress**

It is important to understand the nature of the ultimate employer of these workers: the Department of Energy. DoE is largely a consortium held together in long‐term contracts extending over generations of workers—some extending back to the Manhattan Project—with corporations and corporation‐like entities within universities. Many of the corporations are captive suppliers of materials, services, and components, that is, they have few if any other customers, clients, or markets. Many are actively engaged in technology transfer and personnel exchange with the minimally‐separated private sector. They have often been tied to other, larger multinational corporations. Typically, they maintain Washington “liaison” offices with lobbyists who bypass the Department's headquarters and field staff. They deal directly with the White House, the Congress (especially state delegations and the oversight and appropriations committees) and the “think tanks,” such as specialized science policy and evaluation programs of the National Academy of Sciences funded by the department. There is no other “department” of government like it: an operative amalgam of the private sector.

Progress on workers concerns was often non‐existent, until 1989 when Admiral James D. Watkins became Secretary of Energy and made his first task coping with the special culture of the Department of Energy. This began with a new look at prevention, clinical intervention, and compensation of occupational disease. The pattern of callous indifference to the lives of nuclear weapons workers on the frontline of our nation's defense, reflected in our findings and in the perceptions of workers themselves, verified by the government itself, was broken by the Admiral. The Department of Energy finally had a leader who did not seek unnecessary compromises with the contractors, sub‐contractors, and suppliers that constitute the consortium of enterprises that is the Department, at the cost of the lives of its workers and their families.

Significant improvements were perceived by nuclear weapons workers after the meeting with Admiral Watkins and subsequent meetings of the Secretary's Advisory Committee on Safety and Health. The full costs of occupational disease to workers, to their families and to their communities covered by the compensation laws had never been counted. Medical costs were typically paid from the time that an application for compensation is made, not from the time disease is diagnosed. For claims for cancer, the claimant had to have developed the cancer before he or she applies, and therefore absorbed much of the cost of diagnosis and treatment of the cancer. For some claimants, the cost of treatment bankrupted them.

Of greater import than the dollar costs are the sufferings of these and tens of thousands of other, unrecognized workers and their families, suffering perpetuated by callous policies and slovenly practices that have generated unnecessary death and disease since the very beginning of the Atomic Era.

As Congress attempts to manage the toll of unnecessary risks imposed sometimes blindly and sometimes consciously through cost/benefit analysis, unintended experiments test a new approach to occupational disease compensation: workers' compensation administered not by the insurance industry, but by the federal government.

When first conceived more than a century ago, only one‐time, short‐term incidents for a relatively small number of victims with a limited spectrum of exposure and acute disease attributable to a specific place, practice, or agent were covered.

A fruitful substitute is the discoverable added burden of risk for populations, not fictional probabilities of causation in individual cases. This approach requires more comprehensive industry‐wide studies. “Data, data, and more data,” Irving Selikoff repeatedly insisted, would be needed for multi‐factor systems of compensation.

A century of effort by leading elements of the labor movement to achieve occupational disease compensation reform has led to recognition of the need for new structures of trusted institutions. The moral questions arising from the increased use of genetic markers in medical surveillance and, inevitably, in compensation, highlights the absence of trusted structures for detecting and controlling persisting disease in the nuclear industry. The most common process for the discovery of occupational disease is begun by the victims' families.

NIOSH reports provide a partial history of why the Pantex records of occupational exposures are of limited value. The report on Occupational Internal Dose notes “no routine bioassay program before 1972,”^49^ “little or no tritium bioassay monitoring” in the years 1972 through 1976 or in the year 1984, “only 3% of the files had uranium bioassay results,” “no plutonium or thorium bioassay results were found,”^49^ and “no guarantee that everyone exposed to tritium was monitored.”^49^ The NIOSH report, however, fails to note the post‐1989 watershed effects, illustrated in Table 1.

**Table 1:** Pantex workers monitored and occupational internal dose^49^

Inclusive 15 year periods	Workers monitored for tritium^a^	Workers monitored for uranium	Workers monitored for thorium	Workers monitored for plutonium
1975–1989	4055 [270av/yr]	0	0	0
1990–2004	12,945 [863av/yr]	1515	273	114

^a^ N.B., highest average worker tritium dose years ’75 –’89 = 43.8 (mrem) with a maximum recorded individual dose of 1180 in 1989. Excluding 1989, maximum individual dose is 122. Contrast: 0.2 average dose for years ’90 –’04 with a maximum recorded individual dose of 14 (mrem). The author gratefully acknowledges review by George Gebus, MD, formerly DOE's first Medical Director.

Data reanalyzed from the report on Occupational External Dose^49^ (Table 2), reveal the same effect: radical differences between the 15‐year period 1975 through 1989 and the following 15 years, after significant policy change. More workers monitored, lower exposures found.

**Table 2:** Pantex workers monitored and occupational external dose^49^

Inclusive 15 year periods	Total number of workers monitored	Highest average total dose	Highest total collective dose
1975–1989	13,509 [903 av/yr]	0.25 [rem]	201.19 [person–rem]
1990–2004	41,704 [2780 av/yr]	0.02 [rem]	50.59 [person–rem]

Changes in monitoring instruments and monitoring techniques resulted in changing the bias of the measurements. The dosimetry from 1958 through April 1963 “probably underestimated” neutron doses. From that date through 1976, badge data retrieval services were changed twice.^49^

The NIOSH reports are replete with examples of simple, technically achievable practices that result in radically reduced exposures. The use of lead aprons were “not included in procedures until the mid‐1980s.”^49^

NIOSH staff note that the process of dose reconstruction is “imperfect,” but believe that it is “reasonable,” even in the face of past defective practices: “in the case of internally deposited radio‐nuclides, the regulatory guidelines before the late 1980s did not require that detailed organ doses be calculated [if the quantities deposited were less than recommended maximums]”^50^ The agency's own Health‐Related Energy Research Branch reported in June 2000 a finding that questions the reasonableness of this judgment.^51^ Studying 67,976 female nuclear weapons workers, an independent investigator reported that “recorded doses for external radiation are potentially subject to error because of inconsistent dose monitoring practices … and because certain types of radiation such as neutrons were not measured very well in the past. Confounders such as lifestyle factors, radiation due to medical procedures and other workplace exposures, essential to the dose reconstruction process, “could not be evaluated.”

The pattern of callous indifference to the lives of nuclear weapons workers on the frontline of our nation's defense, reflected in our findings, and in the perceptions of workers themselves, verified by independent investigators and the government itself, was broken in 1989. The Department of Energy finally had a leader who did not seek unnecessary compromises with the contractors, sub‐contractors, and suppliers that constitute the consortium of enterprises that is the Department, at the cost of the lives of its workers and their families.

The result of MTD efforts begun decades ago, the Department of Labor increased payment for medical expenses and wage replacement to former diseased or dead Pantex workers or their families under the Energy Employees Occupational Disease Compensation Act. From January 20, 2012, the effective day of a partially‐positive response to MTD's petition for a Special Exposure Cohort, DOL allowed Pantex claims without radiation dose reconstruction or determining probable cancer causation.

Two classes of workers—those employed in the years 1951 through 1957 and 1984 through 1991—were excluded. MTD reapplied. On October 30, 2013, workers in the ‘84 to ’91 class were admitted to the SEC. MTD called for a special review of the exclusion of workers employed from ‘51 through ’57. The struggle of unions to protect their members begun more than a generation ago was recognized when the Secretary of DHEW granted the petition and initiated the review a year later.

**The Reconsideration**

To avoid conflicts of interest, it is essential that adjudicating bodies and the personnel of structures created to serve them and their petitioners have clear rules that restrain service through declaration of interests and removal of the conflicted from participation. They ought not prevent conscious dialogue on critical values, including rationally necessary moral judgment, guiding conclusions in those constructions of the “as if” universe of discourse that are necessary characteristics of the scientist's molding of scientific methods, and their interpretation.

The original 1985 tables used for determining “probability of causation” fictions for employees covered under the law, as mandated, are revised by the National Cancer Institute (NCI) and the Centers for Disease Control and Prevention (CDC). The product of the original NIH working group was reviewed by a second working group of NCI‐CDC experts charged with revision. Their report^52^ cited a 1984 review by a subcommittee of the National Academy of Sciences/National Research Council.

The NAS/NRC committee objected to NIOSH's use of the term “probability of causation” because that concept “applied to populations and not individuals and could not be interpreted as the probability that a given cancer was caused by a given radiation exposure.”

They recommended using the term, “assigned share” because the computed quantities “are not probabilities in the usual sense and are truly properties of the group to which a person belongs, but in practice are assigned to the person for purposes of compensation.” [Emphasis added.] The NIH and NCI‐CDC working groups were both “sympathetic” to the NAS/NRC views.

The NCI‐CDC group, with some justification, rationalized the “assigned share” concept *as if* it were an actuarial concept and the tables *as if* they were actuarial tables used by insurance companies.^52^

In using actuarial tables, an insurance company applies individual case data gathered by the company's agents and physicians, and does not issue a policy without that data. The problem in using the NIH reconstructions is that the individual case data often does not exist, may never have existed, and often was poorly gathered. Law provides for the establishment of a “Special Exposure Cohort” for these cases.

**The Pattern of Cannibalism: Beryllium**

Someone in the heights of Amarillo awake at 5:30 in the morning of July 16, 1945, looking into the west after a rainy front had left clear skies over the deserts and mountains of the Texas‐New Mexico border, might have seen reflections from the fireball of the first atomic bomb, tested about 300 miles away in Trinity.

At its peak, in 1944, the temporary Manhattan Project of scientists and military planners who produced the bomb at Los Alamos had already employed over 150,000 workers in research, construction and production. Accurate rates of turnover, retirement and replacement are not accessible. It is prudent (using an *heuristic* in scientific lingo) to act as if *millions of nuclear weapons industry workers* then and since served in environmentally‐hazardous laboratories, mines, test and storage sites—and assembly‐disassembly plants like Pantex—from the territories and states of the North Pacific to the New England coast, and from the Canadian to the Mexican border.

Most sites have been abandoned, like the squash court of The University of Chicago, where workers built the uranium pile that enabled Enrico Fermi to demonstrate the chain reaction of splitting atoms at the heart of the bomb. While functions were consolidated or moved, most living workers or their families were left behind without records of exposure or health. The dangers were cloaked in the missions of indentured science and survival in war.

The workers were abandoned to die quietly from multiple factors of disease, including workplace exposures to life‐threatening agents typically unknown to the workers themselves, *even in 1944* well‐known within the biomedical communities. Globally linked through internationally disseminated publications and world‐wide academic exchange, multiple levels of close communication existed, especially among the relatively small number of occupational health professionals directly charged with preventing, treating, observing, and recording the expected effects in the workplace. They operated under wartime strictures of public silence, enforced by their employer: the government of the United States of America. The unions of the nuclear weapons workers, however, were listening.

Unions found their own experts in environmental disease and broke the silence, bringing the plight of nuclear weapons workers and their families to public attention, seeking environmental protection, medical surveillance, and compensation for their members and families to recover the costs of medical treatment and lost wages.

Beryllium is ubiquitous in fossil fuel. As a consequence its gas aerosols may accumulate where the fuel is burned. One investigator surmised that 30% of the population may be predisposed to beryllium sensitivity sufficient to evoke disease. (Saltini C. Recorded comments. Workshops of Department of Energy and Meeting of Beryllium Industry Science Advisory Committee. Washington DC/Santa Fe, NM: Department of Energy, 1993,1994) Thus, workers in most settings—agriculture, food processing, mining, fabrication, distribution, energy generation, construction, communications, disposal, and research—may be subject to exposure to at least low concentrations.

“By April 1946 I had collected 17 cases with three deaths.” Thus wrote Harriet Hardy about the “new” disorder, beryllium disease, found chiefly among women at a fluorescent light bulb factory.^34^ When she delivered her paper before the Massachusetts Medical Society, having failed to censor her, “company officials appeared at the session with a court stenographer who sat in front of me, typing my every word.” The paper^53^ inaugurated decades of research and strife that has no end in sight. It is the special characteristics of this light, solid, and chemically stable metal that encourages the use of beryllium in some form in aerospace, telecommunications, and computer industries, as well as in ceramics, dental alloys, scientific equipment, auto parts, in tool and die making, in addition to the nuclear industries. In 1996, the National Institute of Environmental Health Sciences estimated that “there are approximately 8000 plants with 30,000 workers who may be potentially exposed.”^54^ This estimate, however, was based on a much earlier count that included only heavily‐exposed industrial workers.

In the United States, in 1975, Tony Mazzocchi petitioned on behalf of the Oil, Chemical, and Atomic Workers International Union and the Industrial Union Department of the AFL‐CIO for a replacement of the interim Atomic Energy Commission standard of 1949. Since then, the number of industrial and remediation workers exposed to beryllium dust has increased with its growing applications, while the total number of industrial workers has decreased.^55^ Possibly 800,000 workers in the United States in 1975 were then or previously exposed to beryllium dust, not counting “downstream” users in jewelry and sporting goods.^56^ Government priority focused on the primary beryllium industry and Department of Energy weapons facilities, for whom the prevention of disease, the protection of life itself, is a secondary consideration.

Beryllium's toxic effects have been a concern in Europe since the 1930s.^57^ Delayed effects were encountered in the 1940s by Harriet Hardy at Los Alamos National Laboratory.^53^ But the number of workers with beryllium‐associated disease remains contentious. Almost nothing has been done to look at psychosocial effects: the effects of the devastation of entire families and the economic and structural burden on their communities. In the resulting *anomie,* self‐imposed ignorance is a sentinel symptom of deep‐set social disease, as marked by the union petition for an environmental standard for beryllium in the workplace that had been in an “open docket” at the Occupational Safety and Health Administration for more than forty years. The concentration of effort has not been to prevent sickness, but to propose interventions such as genetic and other medical tests that merely “manage” preventable disease, while reinforcing mores of ancient castes in which the lives of workers are regulated in accordance with lessons learned in the breeding of cattle, to be culled and selected.^58^

Almost from the inception of its use, the identified cases were of acute beryllium disease in uncontrolled environments. After the “silent” period of latency, significant numbers of more difficult to prevent cases of chronic beryllium disease were found. *Chronic beryllium disease, a debilitating and potentially fatal disease, mainly affects the lungs. It almost always is preceded by sensitivity to beryllium exposures less than 0.1 µg/m^3^*. (Information provided by NM Mroz, via personal correspondence with LA Maier). The latency period for the chronic form of the disease can range from months to 30 or more years. About 31% of those found with beryllium sensitivity may progress to chronic beryllium disease after about 4 years.^59^ There is no recommended treatment for sensitivity. The treatment for chronic disease—using corticosteroids—has well‐known adverse side‐effects.

At the Rocky Flats DOE plant, the first identified beryllium‐associated death was a suicide of a young married man with children in his mid‐30s depressed by his dependency and loss of libido reasonably associated with a future of prednisone treatment. After his death, the AFL‐CIO's Workplace Health Fund financially assisted a support group staffed with a psychologist.

The unvented laboratory at Columbia University where in 1940, Enrico Fermi's research assistant became ill was not unusual.^31^ Despite this case and European reports, the Public Health Service had in 1943 declared beryllium to be nontoxic.^60^ A few years later, Harriett Hardy, as medical officer at Los Alamos National Laboratory, encountered more cases of beryllium disease.^34^ Enrico Fermi's widow, Laura Fermi, also had lived in Los Alamos and worked in the laboratory's medical office. As noted here, she was aware of the growing burden of occupational disease associated with the unleashing of nuclear energy.

At a plant processing beryllium for a Department of Energy predecessor agency, measurements were reported in 1946 as high as 4000 µg/m^3^. This was not unique to this one plant. The contamination was widespread. In response, in 1947, an “interim” standard was crafted. Famously, the standard was written in New York City in the back of a taxicab on the back of an envelope by a contractor of the Atomic Energy Commission, Merril Eisenbud, on his way to an emergency meeting at the commission's New York Operations Office.^61^ The standard was Eisenbud's rough estimate of what could be controlled feasibly, within a short period of time, without disruption to production: a 2 µg/m^3^ of air standard for the working day. A 25‐µg limit for short‐term occupational exposures was added later. These limits simply required better housekeeping and equipment maintenance, dust control and proper ventilation. Despite questionable enforcement, it may have eliminated most acute beryllium disease. But the “interim” standard proved to be inadequate for the control of chronic beryllium disease.

Merrill Eisenbud, much of whose life was spent as a consultant to DOE and the beryllium industry, in the final weeks of his life, in 1998, wrote that his standard had not shown “the expected dramatic reductions in the prevalence of CBD.”^62^ He told us that what sick workers had been telling us, for decades, was true.

A 2 µg standard was adopted as a temporary standard by the Occupational Safety and Health Administration in 1971. Attempts by OSHA to upgrade the standard in 1978, supported by National Institute for Occupational Safety and Health recommendations, were stopped by intervention of the Secretary of Energy with the full support of the Office of the President.^63^ After decades the government failed to limit exposure to the feasibly achievable levels at which the disease is not likely to appear.

In 1999, the Department of Energy, for facilities managed by DOE or its contractors, inaugurated the Chronic Beryllium Disease Prevention Program.^64^ The DOE program did not change the environmental limit despite un‐refuted 1977 testimony in an OSHA hearing by the National Institute for Occupational Safety and Health, that a more stringent beryllium standard was feasible: a reduction in exposure to “as low as possible” (one half of a microgram). In 1977, none of three beryllium production facilities surveyed by NIOSH and based on union reports, no DOE facility, was in compliance with the existing 2 µg OSHA standard. (Baier E. Testimony on a Beryllium Standard. OSHA Hearing, Washington, DC, Aug. 19, 1977) Studies within the department by civil servants support a lowering of the standard to one‐tenth of a microgram, based on comparing exposures of workers who developed chronic beryllium disease to those exposed in the general population who did not.^65^

DOE's “prevention” program in great part prevented medical monitoring and removal of sick workers to sheltered workshops or early medical retirement. Facility workers continue to be exposed to beryllium in some 100 different job categories at levels above feasibility known to generate unnecessary disease. By 2004, DOE's medical surveillance program had found more than 700 workers with pathologic sensitivity to beryllium dust and more than 200 workers with manifested chronic beryllium disease among active and former workers: workers occupationally diseased from exposure to just one of a spectrum of toxic agents in their work environment. Just among 13,583 active beryllium‐exposed workers at 21 Department of Energy facilities who volunteered to be screened through December 31, 2007, 236 were found with beryllium sensitivity, the first stage of disease, and 111 with chronic beryllium disease. These were not necessarily long‐time workers: “new cases are being reported among more recent hires.” The DOE reports are forthright. Exposures were elevated in 2003 and 2004. Cases reported in Hanford, Kansas City, Pantex, and Savannah River sites were “inconsistent with low exposure levels being reported” by site management. (2009 Current Beryllium‐Associated Worker Registry Survey. DOE website 6/12/09)

The biological fate of the nuclear worker—“canaries in the coal mine” for workers in every industry—was made clear in Laura Fermi's account of the 1955 Geneva Conference. Participants heard a clear message on risk control and compensation for lost wages and medical expenses from the American delegation, most usefully by Tabershaw and Kleinfeld of New York State's Division of Industrial Hygiene.^66^ The state had oversight of the early stockpiles and processing of uranium.

The Occupational Safety and Health Act of 1970 empowered workers and their peer group leaders, such as union leaders, to participate in implementing positive change in protecting the nation's work environments, a policy adopted by other countries. On every continent, the global labor movement has programs of education enabling participation in the identification and correction of risks in the work environment. Thus scientists need not work alone in bringing science to the task of protecting the health and safety of workers. Passage of the OSHAct enabled this writer, in January 1971, on behalf of the AFL‐CIO, to hand‐deliver the petition for the nation's first permanent occupational health standard, for the control of asbestos dust. It was written by Dr. Selikoff and his closest colleague, engineer William Nicholson, both of New York's Mount Sinai School of Medicine.

Unions have been rewarded in their attempts to enable fruitful answers to ethical questions in the struggle to aid sick or deceased nuclear weapons workers and their families—from mines to disposal—arising in research, medical surveillance, and occupational disease compensation. A letter addressed to this writer, then Special Representative for Nuclear Weapons Workers of the Metal Trades Department, AFL‐CIO dated February 9, 2017, from the Secretary of Health and Human Services, noted final action ending the nearly arbitrary exclusion from compensation of diseased workers whose employment, environmental and medical records supposedly were too incomplete to support precise calculations of compensable risks.

Workers employed at the Pantex nuclear weapons plant in Amarillo, Texas since January 1, 1951 had been excluded from benefits enabled by the Energy Employees Occupational Illness Compensation Program Act of 2000. Their records were being evaluated by government scientists using methods questioned by other government scientists. Unions asked that the methods be evaluated by independent scientists, who found that the prior methods of evaluation were fallacious. The Secretary then acted to ensure better methods.

Union efforts have been recognized for their international scope. To explore risk protection for nuclear weapons workers in Europe, a close colleague of Selikoff, Professor Hans Joachim Woitowitz of Giessen University, a probe was supported by the German Marshall Fund and the U.S. Department of Energy in the *Erzgebirge*. The Wismut mine selected for exploration was the world's deepest uranium mine and had been a primary source of uranium for the production of Russian nuclear weapons. The inspection of the mine revealed an example of the world‐wide scope of conditions consciously rationalized by governments and the professionals they employ.

With the professor's wife, Dr. Rotraude Woitowitz, also a practitioner of occupational medicine, this writer visited a level about a mile below the earth's surface. She donned typical miner's safety equipment and attempted entrance into one of the horizontal channels leading to a mined face of the main uranium vein. She could not reach the face because the channel was too small to accommodate her safety equipment. Clearly, miners attempting to work in the channel faced the same problem.

Returning to the surface, we came to a large room filled with shouting miners. We entered to learn the source of their rage: homes were being constructed for the miners and their families using radioactive mine waste as basic building material, a practice initiated by the former East German government. Having learned of the radiation and toxic dust hazards, the miners were upset about risks to the health of their families. Professor Woitowitz predicted that the new East‐West consolidated German government would not tolerate the practice. He was right. We learned later that the united German government intervened. But we were yet to see still another symptom of global *anomie*.

Reaching the surface, Hans led us to the nearby miners' clinic to show me a room containing large barrels, more than a dozen in number, overflowing with human lungs, the diseased lungs of dead miners.

Our observations in Germany were not unique. American miners and their families in Colorado have endured similar risks. One source of risk are the permissible levels of radiation and dust in the United States, often adopted globally, based on questionable threshold limit values (TLVs) set by committees of the American Conference of Governmental Industrial Hygienists. Their list of TLVs were in the initial set of standards adopted as a temporary measure by the Occupational Safety and Health Administration in 1971. OSHA's initial standards have slowly been upgraded, beginning with asbestos. The agency has never claimed that the threshold limit values describe actual thresholds below which there are no risks. Informally, agency standard‐setters acknowledge “residual risks.”

Globalization of the standards globalizes the good and the bad in our efforts to provide workplace protection and compensation for occupational disease and injury. How may these moral issues be resolved?

## 4 THE FUTURE OF NUCLEAR POWER

**Knut Ringen, DrPH, MHA, MPH**

**Abstract**

All human endeavors entail small and large trade‐offs. Those trade‐offs frequently involve money v. health. Few human endeavors have created more debate about that trade‐off than nuclear power. The idea that uranium could be manipulated to become the most awesome source of energy was hatched immediately after World War II. Harnessing this power for civilian use could help balance out its devastating military uses. Between 1960 and 1980 over 100 commercial reactors were installed (or being installed) and combined they generate 20% of the electricity used in the United State. Then the 1979 Three Mile Island plant criticality, which was compounded by the melt‐down of Chernobyl a few years later, led to serious doubts about the viability of this technology. The 2011 disaster at Fukushima added to the fear.

Nevertheless, it would be wrong to write off nuclear power too fast. First, compare the occupational safety performance of the civilian nuclear fuel cycle (from mining to disposal of spent fuel) to other industries, and also other fuel sources (fossil/hydrocarbon [coal, petroleum natural gas]; hydro; renewables [wind, solar, geothermal]). In the United States, nuclear facilities have improved their safety performance over 90% since 1979. The nuclear power plants operate with injury and illness rates that are 4–5 times lower than in other sources of energy, including during high‐risk outages involving maintenance and repair work.

Based on the nuclear industry's safety performance and capacity to produce electricity, it should be considered an essential component of the national clean energy strategy. However, three vexing questions need to be resolved before nuclear power is embraced more heavily:

- How are the civilian uses of nuclear power kept separate from weapons uses?
- How do we manage the waste?
- How do we prevent complacency, negligence, or fraud in the operation of nuclear plants, given the very significant consequences of nuclear disasters?

**Introduction**

Samuels (elsewhere in this issue) has described how Dr. Selikoff was fond of referring to the “Faustian bargain” we make when we chose between economic interests and safety/health interests. In my discussions with Samuels over the years, we frequently referred to it as the utilitarian's (meaning me) dilemma. Perhaps nothing describes this bargain (or dilemma) better than our mixed feelings about the use of nuclear fuel as a source of energy.

Since World War II, the exploitation of energy for peaceful purposes from the mining, refining, and processing of uranium ore has been debated heavily. This debate has been unusually bifurcated (much like most issues in this political cycle, but much more so than for most other issues historically). You take your side, either for or against; there has been little room for ambiguity or for straddlers.

It is hard to separate out the peaceful uses of uranium from the military uses, and it is also hard to separate out peaceful uses with great direct human benefits (such as medical imaging or industrial radiography) from the more controversial uses of nuclear energy to generate electricity. Interestingly, with the growing urgency created by global climate change and the need to find alternative sources of energy that can divert from reliance on fossil fuels, the debate about whether nuclear power is an “accepted” source of alternative energy has taken on a higher pitch: is it green enough?

Long before the 2011 Fukushima Dai‐ichi nuclear power plant disaster, distrust in nuclear energy had been growing, and this distrust came from two sources. The first is whether nuclear technology, engineering designs and operational procedures are sufficiently robust to control the massive risks involved. The 1979 criticality at the Three Mile Island plant in the US and the 1984 collapse of the Chernobyl plant in the Soviet Union (now Ukraine) drove this distrust to entirely new levels. Second, there were also growing concerns about the cocksureness of energy engineering in general that spilled over into nuclear engineering. A series of great disasters in oil extraction and refinement and in coal operations (from mining disasters to collapses of dams holding vast fly ash waste storage ponds) conflated with risks from nuclear operations: if the less complex fossil fuel operations were not reliable, how could we possibly place our trust in the infinitely more complex and risky nuclear operations?

These uncertainties have resulted in confusing policy responses. Some industrial countries that have relied heavily on nuclear energy (e.g., Germany, Sweden, Japan), appear to be abandoning it. Most of these countries are substituting renewable sources (wind; solar, tidal/wave, etc.), although at least in Japan, the loss of nuclear energy is being backfilled with electricity generated from petroleum and increasingly also coal. Meanwhile, other countries (UK, South Korea, India, China) are embracing nuclear energy at a pace that in some cases (especially China) seems reckless. Traditional nuclear energy power houses such as France and Russia show no inclination to back down from their commitment to this technology even as they struggle to meet increasingly strict safety requirements.

The US is ambiguous about nuclear energy. At its peak, the US operated 104 nuclear power reactors. This number has declined to 95, but because of greater efficiency, output per plant has continued to increase and supply roughly 20% of total energy production.^67^ In 2017 when the last new nuclear reactor to become operational was inaugurated, and it was the relic of a project started in 1973, before the Three Mile Island criticality, that was mothballed in an unfinished state from 1985 to 2008.^68^ It appeared to represent the end of nuclear energy exploitation. Several other plants with similar designs had been abandoned during construction while half‐finished (e.g., Shoreham, NY, Satsop, and Hanford, WA).

Then, 20 years ago, with growing recognition of global warming, the nuclear industry hoped for a “renaissance,” led by companies such as Westinghouse (reactors), GE (turbines), Bechtel (construction), and the Building Trades Unions. Admittedly, the biggest obstacle by then was not safety or liability. The biggest obstacle was getting financing to build new nuclear plants. This was resolved in the Energy Policy Act of 2005, which authorized the government to guarantee loans for construction.^69^ Thereafter, construction of two new plants (each with two reactors) was started, using modernized designs. One, in South Carolina, ended up being abandoned in the middle of construction.^70^ The other, in Waynesborough, GA, will be completed in 2021/22, having taken twice as long to construct as initially projected and costing more than twice as much.^71^ It ended up bankrupting Westinghouse, and almost brought down Toshiba, which had bought Westinghouse.^72^ Long before these plants were completed, the prospect of them making economic sense in the short term had vanished. The nuclear renaissance petered out, not because of safety concerns, but because of a glut of natural gas resulting from fracking.

**The Faustian Bargain**

*The Benefits*

These days a “typical” nuclear power plant produces about 1–1.5 gigawatt (1000–1500 MW) of electricity, which supplies the needs of communities with about 600,000 households. It would take two million acres (an area roughly the size of an average state) covered with solar panels to generate the energy equivalent to the output our nuclear plants, and then, only if they were located in sunny regions with high population density.

**Figure 1:**
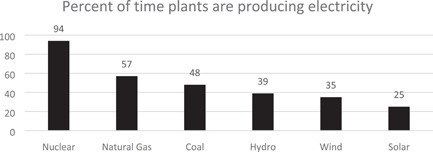
Capacity factor by type of electricity generation, USA, 2019

There is one main reason why nuclear power is still an attractive energy source: its *capacity factor*. Compared to other sources of energy, nuclear fuel wins in the capacity factor race by a long shot. A typical nuclear power plant operates at a capacity factor of about 95%. This means that over its lifetime a nuclear plant will only stop generating energy about 5% of the time. Just as significantly, the lifetime of these plants is proving to be much longer than originally anticipated. While they were originally licensed to operate for 40 years, it now looks like they can easily last twice that long, and many licenses have been extended for an additional 20 years or more. All the while these plants chug along 95% of the time. Compare that to other sources of energy, with much shorter life expectancies and much lower capacity factors: natural gas, 57%; coal, 48%; hydro, 39%; wind, 35%; solar, 25% (see Figure 1).^79^

Because of the longevity and capacity factor of nuclear power plants, it is likely that they are more economical than the other currently known sources of energy. But, reaping that benefit can only be achieved over a very long time, making the high initial investment much more uncertain. Few energy players in the United States seem willing to take that risk.

These days natural gas is a favored alternative because of its low cost and the fact that a gas‐fired power plant is easier to construct than a nuclear plant, both in terms of capital investment and time. It is also a mixed blessing. Electricity generated with natural gas produces about half the particle pollution (about 1 pound of CO_2_ per kilowatt hour) compared to coal (about 2 pounds) or petroleum (1.5–2 pounds).^73^ Even so, gas‐fired plants produce about 50 times as much air pollution as a nuclear plant per unit of electricity generated.^74^

*The Risks*

There are plenty of reasons why nuclear energy should be viewed with caution. First, it is less green than it may seem. The nuclear fuel cycle, from mining to disposing of spent fuel, is filled with energy consumption and creates considerable hazardous waste. To build a nuclear power plant generates huge amounts of CO_2_, and the operation of nuclear power plants also *consumes* energy. The Fukushima criticality did not result from a reactor failure: it resulted from a *power* failure. The offshore earthquake first took out the electrical supply that came from the electrical grid to the power plant. Then, the following tsunami swamped the plant's diesel‐powered back‐up generators.^75^ The electricity supplied to a nuclear power plant is essential to operate the gigantic pumps that drive cooling water to the reactor to keep it from overheating, and for delivering water to the reactor for creating steam to drive the turbines, and finally for pulling the steam from the turbines into condensation facilities that convert the steam back into cold water. In other words, while a nuclear power plant produces massive amounts of energy, it also consumes a fair amount of energy. So, it is quite valid to question how green this energy really is.

Unlike other forms of “green” energy, nuclear‐powered plants produce a lot of waste that is highly radioactive. A typical plant needs to be refueled every 18–24 months. This means that existing fuel rods need to be removed from the reactors and replaced with new ones. This “spent fuel” is still highly reactive. And compared to fossil‐fueled power plants, which also produce a lot of waste, the nuclear waste has a long toxic half‐life. And, when things go wrong in a nuclear plant, it takes forever to remedy the disaster. Fukushima is still highly radioactive, a decade after the disaster there. It has huge ponds filled with radioactive waste that somehow will need to be emptied.

The disposal of nuclear waste has vexed many countries, but especially the US, which has not come up with a permanent solution yet.^76^ Much of this waste can be recycled (more commonly known as “reprocessed”) into new fuel, but only a handful of countries (notably France and Russia) are doing this. A 1960s‐70s pilot recycling program at West Valley, outside Buffalo, New York, ended up being a disaster, with the probable radioactive contamination of thousands of workers being covered up. More recently the US government has held that reprocessing cannot be done in a manner consistent with the Treaty on Nonproliferation of Nuclear Weapons, which was extended indefinitely in 1995.^77^

So, in the absence of solutions, spent fuel is being stored in temporary water pools or in dry cask farms at nuclear plants all over the United States, which raises numerous environmental and security concerns.^78^

**Occupational Risks: Making Nuclear Energy Safer**

**Figure 2:**
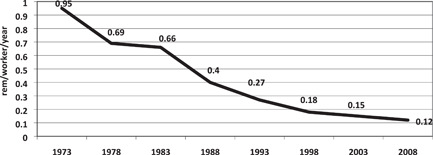
Average annual radiation dose per worker with measured dose

Today, occupational injury and illness rates in nuclear power plants rival those found in low‐risk industries like finance.^76^ It was not always so. At the time of the Three Mile Island disaster, nuclear plants were not much different from other types of engineering and construction sectors, which was not very impressive. Over a 30‐year period since Three Mile Island, however, nuclear energy production went through an amazing transformation in terms of occupational safety and health performance. On every safety performance indicator, including annual collective occupational radiation dose (see Figure 2), nuclear power plants saw improvements of over 90%, while at the same time their capacity factor grew by 60%.^76^

Until the Fukushima disaster, it had been easy to explain past disasters such as Three Mile Island (it operated on a fossil fuel management model) or Chernobyl (lack of preparedness and accountability).^113^ Fukushima resulted not from a lack of being current. It had adopted a vast array of new safeguards developed since 1980. There had in fact been the equivalent of a revolution in the way that the nuclear power industry approached safety.^81^ Still, there were remnants of the arrogance of nuclear engineering even within the Fukushima operation. The International Energy Administration's review of this disaster found that “A major factor that contributed to the accident was the widespread assumption in Japan that its nuclear power plants were so safe that an accident of this magnitude was simply unthinkable. This assumption was accepted by nuclear power plant operators and was not challenged by regulators or by the Government. As a result, Japan was not sufficiently prepared for a severe nuclear accident in March 2011.”^75^

**Occupational Risks: Comparing Nuclear Energy to Other Sectors**

In our 2011 assessment of energy production risks, we concluded: “The relative risks of nuclear energy should be assessed in comparison to the risks of the other energy sources. What has become vividly evident in the past 12 months is that all our major sources of energy—nuclear, coal, and petroleum—require the most careful management of very high risks.”^76^ The tragic disaster at Fukushima happened on March 11, 2011. It capped a year in which, on February 2, a Kleen Energy gas‐fired electricity plant in Connecticut exploded, killing six workers and injuring 50 others; on April 5, 2010, 29 miners were killed in a coal mine explosion at the Big Branch Mine in West Virginia, and on April 20, 2010, 11 oil workers were killed when the Deepwater Horizon oil platform blew up in the Gulf of Mexico.^76^

On the whole, nuclear power generation plants report rates of occupational illnesses and injuries that are favorable compared to all other industries (Table 3) and other sources of electricity generation (Table 4).^114^ During the period 2003–2008, there were no occupational fatalities in nuclear plants, but there were 20 fatalities in hydro plants; 32 in fossil fuel plants; and, five in renewable energy plants. Based on this record we concluded, “…had the nuclear plants operated at the same risk levels as hydro plants they would have experienced a total of 32 fatalities during the period 2003–2008, and if they had operated at the same level of risk as fossil fuel plants, they would have experienced 13 fatalities.”^76^

**Table 3:** Rates of reportable injuries and illnesses for select industries, 2019

Industry	NAICS	Injury and illness rates^a^	
		Recordable	DART
Nuclear Facilities	221,113	0.2	0.1
Finance and Insurance	52	0.8	0.2
Computer and Electronics Mfg	334	1.1	0.6
Pharmaceutical Manufacturing	3254	1.6	1.0
Chemical Manufacturing	325	1.9	1.2
Primary Metal Manufacturing	331	4.4	2.7
Petroleum and Coal Manufacturing	324	1.3	0,7
All Manufacturing	31–33	3.3	2.0
Hospitals	622	5.5	2.2
Educational Services	61	2.0	0.9
***Average for All Private Industry***	‐	**3.6**	**1.8**

*Note*: DART, Cases with days away from work, job restrictions, or transfer.

^a^ Number per 100 FTE workers in industry.

**Table 4:** Rates of reportable injuries and illnesses for different source of electrical power generation, 2019

Industry	NAICS	Injury and illness rates^a^	
		Recordable	DART
Nuclear	221,113	0.2	0.1
Hydro	221,111	2.6	1.7
Fossil fuel (Coal, Petroleum, Natural Gas)	221,112	1.4	0.8
Solar	221,114	0.5	0.5

*Note*: DART, Cases with days away from work, job restrictions or transfer. NAICS, North American Industry Classification System.

^a^ Number per 100 FTE workers in industry.

And, if we look in more detail at the most hazardous work within electricity generation, which is outage maintenance work, the nuclear sector has outperformed other energy sectors with injury and illnesses rates that are 80% or more lower than in fossil fuel, hydro, or renewable sources.^76^

**The Core of Nuclear Safety: Zero Tolerance**

Following Three Mile Island, the US nuclear energy industry concluded it needed to take a zero‐tolerance approach to risk. This sets it apart from all other industries, where cost–benefit decisions are much more prevalent, and also part of the regulatory landscape.

The concept of minimal risk has been an essential element of nuclear safety since health physicists established there is no safe level of human exposure to radionuclides. This led them to adopt the ALARA (“As Low as Reasonably Achievable”) Standard for radiation exposures.

Nuclear generating plants in the United States are regulated by the Nuclear Regulatory Commission, which has vastly more power and resources than any other safety regulatory agencies in the United States. Unlike in general industry, where a workplace may be inspected once in every 30 years or so, each nuclear power plant has at least two resident NRC inspectors that perform daily walk‐throughs of the plants.^80^ And the NRC has a power which no other agency has: it issues, and can revoke, the operating licenses granted to nuclear facility operators.

Moreover, around 1980 the nuclear power operators established a self‐regulatory system, The Institute of Nuclear Power Operators (INPO), which may well have more influence over how nuclear plants are operated than even the NRC.^81^

**Conclusion**

Work in nuclear energy facilities is much safer than work in general industry, or in other sources of energy production, including the renewable energy sources. Nuclear facilities cannot afford even minor mishaps. The owners of these facilities have adopted a system of self‐regulation that is unprecedented, and they abide by a regulatory system that is much stricter than in any other industry.

The Three Mile Island disaster was a watershed that led to the recognition that a general industry approach to safety was not nearly sufficient for nuclear work. This led to the establishment of INPO. In 2011, when the NRC adopted a nuclear safety culture framework,^82^ the transformation of the industry from high risk to exemplary safety was complete.

As a result of these actions, safety indicators for nuclear power plants have declined on average by over 90% since Three Mile Island. Meanwhile, the capacity of nuclear power plants has increased by more than 60%. That is a remarkable transformation.

Does that mean that the industry is out of the woods? Not at all. There remain three great challenges that both nuclear proponents and policy‐makers would like to see disappear by themselves:

How do we keep civilian uses of nuclear power separate from weapons uses? Many countries have so‐called “dual use” reactors that can generate electricity but also produce high grade nuclear materials such as plutonium. The US has one, at Watts Bar, TN, which has been used to produce tritium.^83^ While recent US governments have accepted that to operate the plant for defense purposes would be a violation of the International Non‐proliferation Treaty, we have no assurance that future administrations would see it that way, as is true in other countries with dual‐use reactors.

- How do we manage the waste? It should give no comfort to any proponent of nuclear energy that we have been trying to figure this out without great success (and in the US no success at all) for the past 60 years. As a result, we have vast amounts of spent fuel stored in “temporary” pools or dry casks across the country and vast amounts of mixed chemical and radiological waste stored in huge tank farms.
- How do we prevent complacency or negligence in the operation of nuclear plants? There are lots of near‐miss examples where but for luck the US nuclear plants could have experienced criticalities. The temptation to cut back on scheduled maintenance to improve financial performance has been observed in the past, and without vigilance will occur in the future. Finally, there is a commonplace belief that engineering and operational procedures are fail‐safe, even though there are numerous examples that this belief has been an important causal factor in past disasters and near‐misses, including to an extent in the Fukushima melt‐down, and the response to that disaster.^75^

We need energy, and now more than ever we need clean energy. This entails trade‐offs. For many decades hydropower was considered the safest, cleanest, and least expensive source of energy. It is a paradox that just now, when we recognize we desperately need renewable energy, we also have come to recognize the adverse impact on wildlife of the oldest such source. As a result, hydro dams that have blocked the migration routes of wildlife (especially salmon) are slowly being decommissioned. At the same time, the effects of windmills on bird life is becoming a new challenge. In the absence of technologies to store electricity on a wide scale, or transport electricity over long distances efficiently, hydro, solar, wind, and geothermal energy will continue to face their limited capacity factors, and a growing sense that many people don't want these plants in their local vicinity.

No matter which source of energy, there are trade‐offs. Natural gas is the current go‐to solution for new energy. It is cheap, plentiful, and available just about everywhere. It has also created a vast fracking industry with its own safety problems. Does that make it right, or, wrong? It depends on who you ask. It depends on what kind of Faustus (or utilitarian) you are. I, for one, am not yet willing to write off nuclear energy. The benefits are simply too great.

## 5 GLOBAL WARMING

**William N. Rom, MD, MPH**

**Abstract**

Irving J. Selikoff MD began his academic medical career studying tuberculosis treatments followed by researching the increased risk of asbestos workers for asbestosis, lung cancer and mesothelioma. He founded the Environmental Sciences Laboratory at Mt Sinai with a mission beyond asbestos to styrene and PCBs in the workplace, gases like SO_2_ in paper and pulp mills, and environmental exposures like polybrominated biphenyls in the Michigan farmers. He would have embraced research on greenhouse gases from anthropogenic sources causing global warming. A 1.3°C increase in global average temperature due to anthropogenic (man‐made) fossil fuel consumption has occurred over the past 50 years. Greenhouse gas production, including CO_2_ and methane, has increased by over 40%, trapping heat at the earth's surface and in the oceans. Rapid phase‐out of fossil fuels with increases in efficiency, wind, solar, and modular nuclear energy can ameliorate the carbon pollution. Wind provides 7% of U.S. electricity generation and produces over 100 GW of our 1200 GW energy supply. An increase to 600 GW (half of the energy supply) over the next 15 years is possible at ~20 GW/yr and 50 GW of off‐shore wind. The already extant 115,000 jobs and 25,000 factory jobs in wind turbine construction would see a dramatic increase. Solar photovoltaic panel production could be incentivized to increase from 1% to 2% to 10‐fold over 15 yr for electrification of homes and commercial buildings. Solar farms could produce cheap renewable energy in tandem with wind turbines and battery storage to replace coal and natural gas power plants. Electrification of transportation, stoves, heat pumps, and the steel and cement industries could meet carbon‐neutral goals by 2050.

**Introduction to Climate Science**

**Figure 3:**
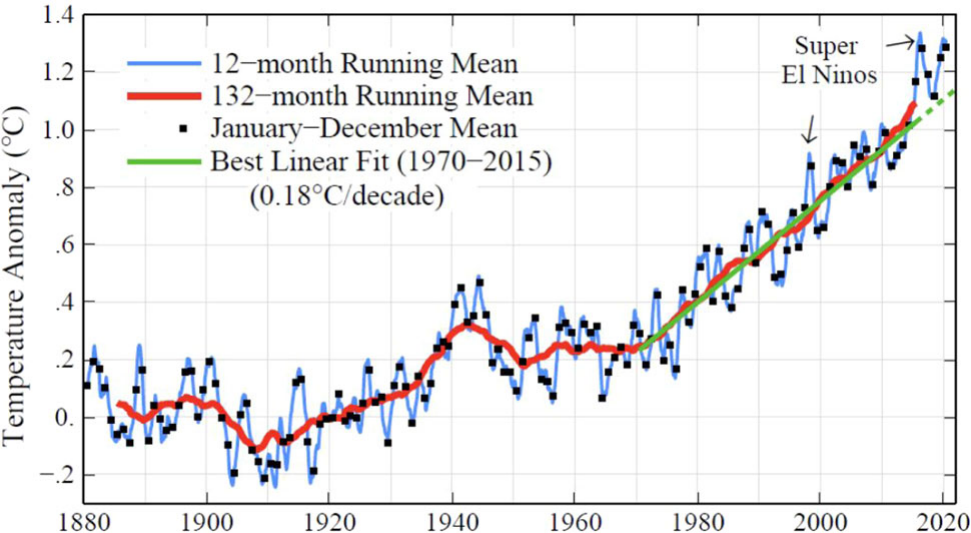
Global surface temperature relative to 1880‐1920 Mean. 2020 12‐month running mean reaches 1.3 degrees C

Svante Arrhenius in the late 19th century demonstrated that measurements of global greenhouse gases and temperature seemed to show a complementary effect. As fossil fuel consumption increased CO_2_ in the atmosphere, he predicted a planetary greenhouse effect where the CO_2_ layer reflected infrared radiation trapping heat and the planet Earth would be decisively warming. In 1988, James Hansen of the NASA Goddard Institute in New York and affiliated with the Earth Institute of Columbia University testified in Congress that global warming was now occurring, that is, a real phenomenon.^84^, ^85^, ^86^, ^87^, ^88^ Senator, later Vice‐President, Al Gore took up the cause and was awarded the Nobel Peace Prize along with the Intergovernmental Panel on Climate Change for the documentary “An Inconvenient Truth” and the IPCC Third Report stating “Global Warming was Unequivocal,” respectively. The global mean temperature for 2016 and 2020 tied for the warmest years on record and 19 of the 20 warmest years on record have all occurred since 2000. (Figure 3, with permission of James Hansen).

The mean temperature was now 1.3°C above the preindustrial baseline. This correlated with measurements of 419 ppm CO_2_ on the summit of Mauna Loa in Hawaii, an increasing trend known as the Keeling Curve. Charles Keeling began CO_2_ measurements in 1958 as part of the International Geophysical Year. He chose Mauna Loa since its summit was almost 14,000 feet and should be representative of an unpolluted atmosphere. Initial CO_2_ measurements in 1958 were 310 ppm, and have increased almost 40% since measurements began. Keeling noted that there was a jagged pattern to the increase, with CO_2_ falling in the northern hemispheric spring when leaves sprouted and took up CO_2_, and that CO_2_ increased in the fall after leaves fell off the trees. The southern hemisphere made no difference to this pattern since most of the earth's land mass and trees are in the northern hemisphere. He also noted that the increase each year was accelerating from 1 ppm initially to almost 3 ppm more recently. He correlated this increase to fossil fuel combustion that increased emitted CO_2_ from a few billion tons in the 1960s to over 36 billion tons of CO_2_ annually across the globe.^87^, ^88^ Another 5 billion tons of CO_2_ is emitted from deforestation and agriculture (cows burping methane, nitrous oxide released from rice paddies, etc).

The United States has been the greatest emitter historically—almost half—with much of the remainder coming from England, Russia, and Japan. For the past decade, China has been the lead emitter. Selikoff would have noted the immoral role of the United States being the major emitter and doing little to mitigate global warming. He would have decried our long support for fossil fuel energy, especially in the developing world instead of renewable energy.

The planet Earth is in a “sweet spot” between Venus where the atmospheric concentration of CO_2_ is dense causing such a greenhouse effect that the surface temperature is 475°C, and Mars where the atmosphere is much less dense (although the percent of CO_2_ is the same) causing the surface temperature to be minus 60°C. It is amazing that the Earth is mostly nitrogen (79%) and oxygen (20%) with CO_2_ only 0.04% of the atmosphere; interestingly, the greenhouse layer is so tightly regulated by the carbon cycle that most CO_2_ emissions are taken up by the ocean (30%) and forests, soil, and rocks, leaving only parts per million for the atmosphere. We have learned from Antarctic ice cores drilled up to 3000–4000 m into the icecap that tiny bubbles within them contain a record of the earth's CO_2_ and temperature going back 800,000 years. CO_2_ measurements in the tiny bubbles never exceed 280 ppm until recently showing that we have never had such an increase as the current Anthropocene (human‐dominated environment). Isotopic measurements of water used as a proxy for temperature indicates that whenever CO_2_ increased, temperature according to the ice record also increased. Paleo‐ecologists going back in the fossil record millions of years have found periods of 2°C warming due to increased CO_2_ which correlated with more than 10 m of sea level rise above current levels.

**Health Consequences from Global Warming**

There are consequences from global warming that we can observe in the present, and with the knowledge that worse is yet to come.^86^, ^87^, ^88^ There are heat waves where hundreds and thousands perish due to heat stroke, cardiovascular, and pulmonary diseases, especially the elderly and infants where there are few if any air conditioning units. There will be more periods in the summer where extreme temperatures will persist for days and weeks on end. Higher temperatures increase evaporation of water into the atmosphere, and deluges of rain causing rivers to flood will increase. Conversely areas of low precipitation will have droughts adversely affecting agriculture in California's Central Valley and the Great Plains and Upper Midwest where irrigation is intensive. Higher temperatures will decrease maize, rice, and wheat yield by about 10%, and protein, zinc, and B vitamins in crops will all be reduced. Food insecurity will be exacerbated, and over several years will result in net migration. Migrants will cause conflict as they cross borders. This has already happened in the highlands of Guatemala and Honduras where farmers and their families migrate to the U.S. southern border seeking asylum and protection. The farmers of Syria migrated to the cities after years of drought and were met with violence from troops of the dictator Assad. Farmers from Africa's Sahel are suffering years of drought and migrate north trying to reach Europe. Increased precipitation will cause hurricanes and cyclones to intensify and stall over land inundating populated areas like New Orleans, Houston, New York City, the Philippines, Mozambique, and Puerto Rico.^89^ These storms cause a huge loss of life during and after the event, and cost hundreds of billions of dollars in damages causing many insurers to take pause in insuring coastal housing.

Burning fossil fuels cause tremendous air pollution, with emission of fine particulate matter, nitrogen oxides, and ozone formation. WHO estimates 3.5 million persons die from outdoor pollution annually, with the greatest burden in India and China.^87^, ^88^, ^90^ Burning biomass, such as cattle dung, for heat and cooking in houses without proper chimneys is an additional problem affecting most developing countries. Women and their children are at particular risk for chronic obstructive pulmonary disease and respiratory bronchiolitis, respectively, since they have the burden of cooking. WHO estimates another 3.5 million persons die from these types of indoor air pollution annually.^87^, ^88^ Intensifying current air pollution is the increased frequency and extent of forest fires. Forests are increasingly susceptible to rapidly‐moving crown fires as drought makes trees increasingly dry, and insects such as the bark beetle kill entire forests because the winters are now too warm to kill their larvae. As with other types of air pollution, the particulate matter and volatile organic chemicals released by fires are hazardous to health; there is a rapid spike in emergency department visits downwind from fires due to the extensive smoke. Respiratory illnesses such as pneumonia, exacerbations of chronic obstructive pulmonary disease, asthma attacks, and heart attacks and dysrhythmias all increase as a consequence of airborne pollutants released from fires.

**Ecological Consequences of Global Warming.**

Over 90% of the heat from global warming is absorbed in the oceans. The increased heat is toxic for the world's coral reefs that suffer “bleaching” with heat events.^84^, ^85^, ^86^, ^87^, ^88^ Bleaching occurs when the symbiotic algae leave the coral causing it to lose their color and turn white as well as cutting off the source of photosynthetic nutrients for the coral. As the ocean warms, coral reefs fail to recover from bleaching events, and eventually die. More than a third of fish of the world spend part of their lives in coral reefs, and their loss will severely impact fisheries. Many of the world's poor fish off their shores among the coral reefs and these events intensify food insecurity.

Global warming will cause sea level rise from both thermal expansion and melting glaciers. The temperate glaciers are rapidly disappearing; for example, Mount Kilimanjaro's ice coverage has declined from 11 to 1–2 km^2^ over the past century. The glaciers are disappearing in Glacier National Park which will cause the park to be glacier‐free by 2050. Glaciers' runoff power hydroelectric dams in alpine countries such as Switzerland, and provide essential drinking water to huge cities like Lima, Peru. Most important are the glaciers in Greenland and Antarctica that are advancing due to warmer sea water melting them from below as well as surface melt. Their melting could cause sea level to rise dramatically over the forthcoming decades. Estimates of future sea level rise have increased from 1 to 2 m by 2100 depending on whether we follow business‐as‐usual practices in fossil fuel emissions or pursue more aggressive mitigation efforts. A 2‐m rise would jeopardize Miami, New Orleans and New York City; the Florida Everglades would be inundated and most of Miami would be under water.

**Mortality from Climate Change**

Mortality from climate change is estimated by WHO to be approximately 150,000 deaths annually which is expected to almost double after 2030 without aggressive mitigation efforts. Estimates after 2050 to 2100 suggest 155 million deaths could be avoided if air pollution controls were incentivized.^84^, ^85^, ^86^, ^87^, ^88^ Another estimate suggested 106 million deaths would occur 2018–2100 globally under the business‐as‐usual scenario where the global mean temperature rise would exceed 3°C. The “Climate Crisis” would be considered by Selikoff to be a moral crisis since the burden of the adverse effects are borne by poorer and developing countries in the southern hemisphere. Global economic output will be reduced 23% by 2100. The second aspect of the moral crisis is the burden we are placing on our young people and offspring to deal with the adverse effects of climate change in the future where the economic costs will be multiplicatively greater and mortality more severe. Third, Selikoff would consider a moral crisis sparked by “climate deniers” delaying our response to this global emergency and increasing its risk and cost. Lastly, Selikoff would decry the moral crisis for biodiversity as the Anthropocene triggers a possible “sixth extinction” where the increased heat and industrial and agricultural development make Earth uninhabitable for thousands of species.

**Trade Unions and Occupational and Environmental Protection**

Unions have been the backbone of the American middle class, arguing for a decent livable wage, health benefits, and safe and healthy workplaces. They were instrumental in providing support to pass the Occupational Safety and Health Act of 1970 (OSHAct). The OSHAct created the Occupational Safety and Health Administration in the Department of Labor that set workplace standards, and employed staff to inspect workplaces to ensure a safe working environment. They formulated a general duty clause for a safe and healthy workplace and began the process of establishing standards for common hazards such as asbestos, silica, beryllium, benzene, SO_2_, and solvents such as trichlorethylene. A National Institute for Occupational Safety and Health (NIOSH) was also established and placed in the Centers for Disease Control rather than the National Institutes of Health since they had the charge of recommending exposure levels to OSHA, a quasi‐regulatory function. Under CDC, NIOSH was spread among multiple sites including Atlanta CDC headquarters, the Washington D.C. HHS central office, Cincinnati (Epidemiology and Training) and Morgantown (Respiratory Diseases especially the Coal Miner's Surveillance Programs, and Toxicology). NIOSH dispenses extramural research grants and established interdisciplinary training programs, the Educational and Research Centers located in each HHS region of the country. NIOSH performed in‐house research on occupational diseases including large population studies, and health hazard evaluations that could be requested (anonymously) by any worker.

The OSHAct also established an advisory committee to provide advice to both NIOSH and OSHA. Also, in 1970 President Nixon established the Environmental Protection Agency to regulate pesticides, control air and water pollution, and protect the US population from radiation hazards. The Clean Air Act of 1970 was far‐reaching in that it required air pollution standards to protect human health with an adequate margin of safety including for vulnerable persons (those with chronic disease and the elderly) and very young populations.

The Clean Air Act required EPA to establish the National Ambient Air Quality Standards for six exposures: particulates, SO_2_, ozone, NO_2_, lead, and carbon monoxide. A Clean Air Scientific Advisory Committee was to advise the EPA Administrator on the extent and effects of pollutant exposure to ensure a level of safety for health. The Clean Air Act required that individual states develop a permit system that each polluter was not to exceed, to keep within the limits of the overall primary standards. States would have several challenges, since power plant emissions travel across state borders and mobile transport was very difficult to regulate. To meet this challenge, California, which had some of the most severe air pollution hazards, was allowed extra leeway in their regulation. Over the first 45 years of the EPA there was a significant improvement in air quality with most pollutants reduced over 90% at a benefit‐to‐cost ratio that approached 30:1. The Clean Water Act of 1972 gave the EPA similar authority to regulate discharges into navigable waters of the United States. EPA provided funding to distribute to localities to upgrade sewage treatment. Providing at least secondary treatment was important in cleaning the nation's waterways while some communities also required tertiary treatment since algae had become a significant problem from untreated or partially treated sewage over the years.

Importantly, labor unions supported both the creation of OSHA and the EPA, joining working men and women with environmentalists in a common cause. Environmentalists were clearly in favor of a clean and healthy workplace, and workers would gain clean air, clean water, and parks that they could enjoy with their families. Wilderness areas grew from 9 million acres to over 110 million acres over the half‐century of the Wilderness Act. Environmentalists joined labor unions as a constituency for ample minimum wage and health benefits. This became a grand coalition in creating programs that benefited both groups: the Land and Water Conservation Fund that was recently re‐authorized and has funded more than 40,000 recreational projects remains a prime example.

Union membership has been in decline over the past 50 years due to de‐industrialization of traditional manufacturing regions and lack of union organizing in new trades such as the service and technology and communications industries. Corporations have obtained lower labor costs by moving operations to right‐to‐work states that curtail union organizing, and more drastically, have taken manufacturing to China, Southeast Asia, Mexico, and Latin America. These countries have been training skilled labor and developing supply chains that replaced those in the continental United States. Corporations have been innovative as well. Plant closures have left many workers without traditionally high‐paying un‐ionized manufacturing jobs, forcing them into low‐wage service jobs, and requiring them, in many instances, to work at least two jobs to stay afloat financially. This makes them vulnerable to demagogues and political con artists promising a return to the past. Right‐wing politicians' belief in tax cuts for the rich (again in the guise of stimulating job creation and growth) then places pressure on the government programs that support workers, from grants for education, unemployment insurance, Medicaid, the Affordable Care Act, and many others. Trumpism is the culmination of this fraud, providing disenchanted workers with a false sense of agency, destroying government programs by “draining the swamp,” attacking the news media as “enemies of the people,” and disarming the values underlying American democracy.^90^

**Unions and Renewable Energy**

Enter the 2018 midterm election with a change in control of the U.S. House of Representatives and introduction of the progressive Democrats' Green New Deal. This aspiration has three underlying tenets: work toward 100% clean energy by 2030; provision of universal health care; and a guarantee of a job for all working Americans. In October 2018, the United Nations' Intergovernmental Panel on Climate Change reported that we have only 12 years to aggressively mitigate global warming, by limiting the increase in global mean temperature to 1.5°C to avoid the worst effects of climate change in the future.^85^ Of course, accomplishing 100% clean energy in 10 years is a very heavy lift, but it is aspirational although President Biden established 2035 as the target to achieve fully renewable clean electricity and 2050 as the target for net‐zero carbon release. Already five states are on track to achieve this legally (California, Hawaii, New Mexico, Washington, and Nevada) with New York and Massachusetts heading in this direction.

First, to achieve the 2050 goal we will have to increase renewable energy to replace fossil fuels in the electricity‐generating sector. The Sierra Club, with over $150 million from Bloomberg philanthropies, has mobilized its members to shut down 278 coal‐fired power plants. This is half of the coal‐fired generating capacity in the United States. More importantly, wind and solar energy is cost‐competitive to coal, and economics is, and will, drive these closures. The most important reason for unions to support the Green New Deal is jobs. Jobs are flowing toward the renewables, and they represent over 300 job categories that should be un‐ionized; in contrast, jobs are fleeing coal mining, coal transport, and coal‐fired power plants. There are three million clean energy jobs at the end of 2020; these are in energy efficiency, solar, wind, and electric vehicle manufacturing. Clean energy jobs are 40% overall of the energy workforce. The solar industry employs 375,000 workers^91^ which is double the coal industry employment in 2019, and produces 1.7% of the nation's electricity. Natural gas employs 7.7% more workers, but 80% of those are engaged in producing fuel rather than electricity. The natural gas, oil, and coal industries employed only 187,117 workers in 2017.^91^ Wind industry employs ~110,000 workers and hydropower employs 65,000 workers.^91^ Now there are fewer than 50,000 coal miners, down from 823,000 in the peak year of 1923. In West Virginia there were 12,555 jobs in underground coal mines and 2935 in surface mines. Electricians, plumbers, wind turbine technicians, solar technicians, construction workers in the renewable energy fields, and many others are potential recruits for expanding union membership. The large coal strip mines in Wyoming and Montana have been transporting coal to western ports for shipment to China; environmentalists have been protesting successfully on coal shipment to these port terminals, preventing expansion, blocking new construction of terminal ports, and applying pressure on existing export terminals. The wind industry produces 105 gigawatts of electrical power (7% of U.S. electricity), and expects to reach 20% of US electricity output and employ 380,000 workers by 2030. Work as a wind turbine technician is the fastest‐growing occupation in the U.S. according to the U.S. Bureau of Labor Statistics. In promoting renewables, it is imperative to re‐train workers in oil, gas, and coal extraction in the technical aspects of renewables and locate as many jobs in displaced fossil‐fuel‐driven communities as possible. Development of renewable energy transport will need additional high voltage direct current (HVDC) lines, and this critical infrastructure will require many more jobs. Off‐shore wind has a huge advantage of proximity to large cities on the East Coast for ease of transport and fewer requirements for crossing private lands.

Second, transportation emits tons of air pollutants, and traffic congestion impairs gross domestic product as highways become increasingly overcrowded. Beginning in the early years of this decade, we may have an explosion in electric vehicles with less vehicle ownership and artificial intelligence driving autonomous vehicles. Millennials show the least interest in owning cars, and use ride‐sharing to commute short distances. They look askance at the cost of purchasing a new vehicle, plus insurance, parking, and maintenance. The negatives are beginning to outweigh the positives. The future for automobile manufacturers, auto dealers, gas stations, mechanics and truck drivers appears bleak, with as many as 10 million jobs about to disappear. This will be hugely disruptive. On the plus side will be reduction in carbon pollution, the reduction in particulate matter and nitrogen oxides, reduced congestion in cities and highways, and reduction in sprawl. The growth in jobs for producing batteries, building electric cars, developing artificial intelligence and expanding computer power, re‐building railroads and infrastructure will need to keep pace with the loss of jobs in making and maintaining the internal combustion engine. Note that the internal combustion engine has 2000 moving parts and the electric automobile has fewer than 20 moving parts. Building out electric charging stations at interstate rest stops and converting gas stations to electric super‐charging stations will be necessary. Charging stations could take advantage of renewable energy by placing a solar farm or wind turbine in their vicinity. Building storage batteries will require more lithium and precious metal mining and processing including nickel and cobalt. These processes will require research into new technologies that will not pollute the environment.

Third, the National Oceanic and Atmospheric Administration (NOAA) published a road map in 2016 to achieve a cost‐optimized single electrical power system for the US by 2030.^92^ Their report indicates that the US can reduce CO_2_ emissions from the electricity sector by about 80% at approximately the same cost of electricity as in 2012. Using the low‐cost renewable energy/high‐cost natural gas scenario (2006–2008), US power consumers could save an estimated $47 billion annually with a national electrical power system versus a regionally divided one. This amounts to almost three times the cost of an HVDC transmission network per year. Their report envisioned 2030 as a time when wind produces 38% of the electricity (523 GW or about a 6‐fold increase), 17% by solar photovoltaic (371 GW or about 62‐fold increase), 21% natural gas, nuclear 15%, and hydropower 9%. The land requirement would be 460 km^2^ for wind turbines and 6110 km^2^ for solar photovoltaic farms or 0.08% of the US land mass. The need for water would be reduced by 65% due to fewer steam turbines. They admit that there are formidable challenges: integration of variable generators; changes to the existing regulatory commercial and legal frameworks; and investments in the HVDC network and new power plants. They note that if the electricity sector were de‐carbonized, there would be good prospects that electrical vehicles, heat pumps, and other electricity‐based technologies would similarly reduce CO_2_ across the energy sector. They compared the clean energy effort to transitions in the past including the transcontinental railroad of the nineteenth century, and the interstate highway system of the 20th century.^92^

**Cities and Renewable Energy**

New York City has been a leader among 119 cities across America that have embraced a clean energy future. Most of New York's carbon pollution comes from its buildings. The City Council passed a resolution that would cut carbon pollution by 26% by 2030 (40% compared to 2005 levels by 2030). More than two‐thirds of carbon pollution comes from buildings, and luxury towers representing 2% of the buildings emit half of the CO_2_ pollution. New York City also has many large apartments that burn heating oil, while most of the recently constructed apartments, businesses, academic institutions, and hospitals burn natural gas. Cooking stoves are heavily committed to natural gas, illustrating the opportunities to convert to electric or induction stoves and clean electricity fronted by off‐shore wind, for example. Changing to LED lighting in thousands of apartments would increase energy efficiency.

**States, Unions, and Renewable Energy**

The Apollo Alliance two decades ago brought together unions, business and environmental organizations together to coordinate efforts for the new clean economy; more recently the Blue Green Alliance brought 13 unions and enviros together (utility workers, steelworkers, painters, plumbers, service workers, teachers) and environmental groups (Sierra, Natural Resources Defense Council, Environmental Defense Fund, League of Conservation Voters) pledging that working people are to be front and center as we create a new economy.^93^ There are several ways to achieve this: one is to ensure strong labor standards to government programs and contracts, another is to fund union training programs as apprentices as wind turbine technician, solar panel installer, etc., and third to pass legislation to enable employees to organize union membership without employers' interference.^93^ Unions have declined from 30% in 1970 to 10% of the workforce in 2020 with only 6% un‐ionized in the renewables energy sector. In 2019 Washington State enacted the Clean Energy Transformation Act that tied labor standards to tax incentives for clean energy development. These standards included apprenticeship utilization, prevailing wage, local hire, and the use of Project Labor and Community Benefits Agreements that cover multiple trades and involve community organizations.^93^ A large wind farm called Rattlesnake Flats in Washington State was developed on this basis. New York's Climate Leadership and Community Protection Act requires clean energy developers to pay the prevailing wage and utilize Project Labor Agreements, as well as to use American‐made steel. New Jersey has a regulation that solar installers with more than 1 MW pay the prevailing wage and use Project Labor Agreements to receive Renewable Energy Credits. Virginia's Clean Economy Act directs the utilities in building over 5000 MW of wind energy to utilize Project Labor Agreements and hire and apprentice locals, veterans, and traditionally economically disadvantaged indivisuals. The Protect the Right to Organize Act that recently passed the US House of Representatives would help in organizing union membership and require those not in unions to pay membership fees. Unions have been shown to increase wages and benefits for those not in unions by almost 30%, and strengthens the middle class and reduces inequality.

Carbon capture and sequestration is part of the equation that would solve carbon pollution in the steel and cement industries, and some newer natural gas‐fired and coal‐fired power plants. Carbon capture may be necessary to attract political agreements on climate change and preserve union jobs. Currently, the San Juan plant in New Mexico and Great River's Coal Creek coal‐fired power plants in North Dakota are scheduled for closure, but new companies have purchased these power plants to install carbon capture technology. This will preserve union jobs in the power plants and coal mining, and develop new jobs in carbon capture and sequestration. Both of these coal‐fired power plants are losing money and will depend on billions of dollars in government subsidies to develop carbon capture. Importantly, bipartisan political agreements that will favor renewables such as wind and solar, electric vehicle charging stations, a clean electricity standard, resiliency in the grid and HVDC transmission lines, and so forth will need carbon capture and sequestration to garner enough political support to pass in the US Congress.

There will always be a need for collective bargaining to protect the gains of workers, while an alliance with the environmental movement provides strength to survive and prosper among the dislocations as we move toward a renewable energy future.

## 6 WORLDWIDE CHANGES IN OCCUPATIONAL HEALTH PROVISION OVER THE PAST HALF CENTURY

**Arthur L. Frank, MD, PhD**

**Abstract**

The provision of occupational medicine services has changed little over the past half‐century or longer. In some ways, it has retrogressed. As a leader in the field of occupational medicine, Dr. Irving J. Selikoff not only contributed to the scientific literature but established a training program and led international efforts to promote occupational health. More needs to be done to make occupational and environmental health a more significant part of medical education.

**Origins**

The origins of what we now call occupational medicine go back hundreds of years. In 1543 Paracelsus commented that “The dose makes the poison.” Little was added to this view, or to the basics of medicine as noted by Hippocrates centuries before, until the work of the Father of Occupational Medicine, Bernadino Ramazzini, who in 1700 published the first edition of *De Morbis Artificum Diatriba*.^94^ In that book he discussed not only dozens of work‐related illnesses but added to the medical questioning of patients an inquiry of what individuals did for a living. Unfortunately, in over 300 years of medical practice since, that inquiry has not entered into the mainstream of medical care or investigation.

Following this initial work in Italy, subsequent occupational exposures in France regarding lead poisoning, written about by Tanquerel des Planches,^95^ work by Thackrah in England in connection with the Industrial Revolution, and the work in Eastern Europe regarding radiation exposure in mines leading to lung cancer by Harting and Hesse.^40^ In the United States, occupational medicine did not become a serious discipline for many years thereafter and has never become mainstream. The railroad industry employed physicians and in the late 1890s there were texts on “Railroad Surgery,” and finally in the early 20th century Dr. Alice Hamilton, the Mother of Occupational Medicine in the United States took this field into a somewhat more prominent position. Dr. Hamilton became the first woman ever appointed to a faculty position at Harvard, at the School of Public Health, although her gender prevented her from using the faculty club, getting football tickets, and caused her to be uninvited to the annual Harvard graduation. Following her introduction of occupational medicine into the American medical landscape, she and others began to publish in this field.^96^

Traditionally, in the United States, most occupational medicine physicians work in corporate industrial settings. Large companies had departments of occupational medicine, sometimes closely affiliated with industrial hygiene and safety activities. One estimate is that for every 5000 workers in a company there should be one occupational physician. A second setting for occupational physicians has been in the world of academic medicine, and in the 1960s there were about two dozen training programs to educate and train additional occupational physicians. With the advent of NIOSH and its funding of training programs, as well as additional funding through a scholarship fund set up through the American College of Occupational and Environmental Medicine (ACOEM), about forty programs were available for training towards the end of the 20th century. ACOEM had not added the term “environmental” until 1992, reflecting the very recent addition of the appreciation of environmental factors in medicine. Unfortunately, with no increase in funding over time, this medical specialty with its great shortfall of physicians available compared to need, has lost many training programs and there are now about the same number as existed some 50 or 60 years ago.

In the United States, in addition to corporate and academic positions, one will find private practices of occupational medicine, occupational medicine services given at walk‐in type clinics, some NGOs, such as labor unions, employing physicians trained in occupational medicine, and generally a small number found in various governmental settings including military settings.

**The Teaching of Occupational Medicine in the USA**

One of the leading occupational physicians in the United States, Dr. Irving J. Selikoff, who served as head of the Environmental Sciences Laboratory at the Mount Sinai Hospital and then at the Mount Sinai School of Medicine when it opened in 1968 with students, felt it important to train additional occupational physicians. As a professor at Mount Sinai, he started an occupational medicine residency, which was complemented by a general preventive medicine residency elsewhere in what was then the Department of Community Medicine. It was in this setting that the author of this paper received his training, and then had the privilege of overseeing the two residency programs upon his finishing his own training. It was also at Mount Sinai Hospital that the author learned of the continuing resistance to incorporate an occupational and environmental medicine history into routine medical care. Having trained also in internal medicine, the author and Dr. Selikoff, who had trained as a pulmonologist, put together what was called “the 95% solution” which meant the one‐page form that would, in a 5‐min bit of history taking, collect some 95% of relevant occupational and environmental exposure information that might assist in the diagnosis and care of patients.

A form was created,^97^ approved for testing by the appropriate committee at Mount Sinai, and was then “mandated” to be used for all new internal medicine admissions to the Mount Sinai Hospital on one of the medical ward floors. Simply put, this procedure was not adhered to, and after a period of several months the experiment and the potential use of this form were abandoned. Few had bothered to fill it out.

This is not a surprising outcome to those who understood the history of teaching of occupational medicine in medical education. Levy, and others, published a series of papers^98^, ^99^, ^100^ documenting how little medical education at any level included the teaching of occupational medicine. Part of the problem was a dearth of occupational physicians who were on medical school faculties, with a considerable proportion of medical schools having no one trained in occupational medicine. When first evaluated, of 112 medical schools, 92 responded; required teaching occurred only at 28 schools and the average amount of time in a 4‐year medical curriculum was 4 h. Traditionally, this teaching was included in such fields as basic pathology, or for lead poisoning, might be taught in pediatrics. A 5‐year follow‐up from 111 of the then 127 medical schools found that only two‐thirds did any teaching, and the amount of time was still 4 h. Another follow‐up some 4 years later found the same proportion of 115 schools responding teaching occupational medicine principles, which had now increased to 6 h. This author, when responsible for the curriculum of a Department of Preventive Medicine and Environmental Health at one American medical school, spent some 8 h of a 40‐h curriculum devoted to occupational and environmental health teaching. Clearly, this is not a field of medicine that has ever been successfully taught in American medical schools.

This fact is somewhat remarkable, since about half of the American population is engaged in some form of employment, and related potential exposures, and everyone in America, and in every country, lives in the generic environment that includes exposures to potential hazards in air, water, food, and elsewhere.

One might ask why such a situation may exist. There are clearly a number of reasons, including the traditional fights within medical schools for curriculum time, and the clinical departments carrying more sway than departments of preventive medicine, some of which are relegated (in the eyes of clinical chairs) to the status of a basic science department. Medicine, as a profession, is extraordinarily conservative in its ability to modify or change. Over time some changes have come, such as now finding the majority of medical students, some 52%, are female compared to 50 years ago when as a student my class with 15% women was considered an extreme outlier at the time with such a “high” number. Traditionally, medical students came from upper‐middle‐class and upper‐class families, and often the sons of professionals. Therefore, these physicians in training had little in common with, nor understood, the day‐to‐day lives and exposures of many working people. Personal data documented how poorly medical charts reflected potential workplace or significant environmental exposures, both some 50 years ago and now—as someone who still reviews charts regularly, I see how little has changed. In a chart review undertaken by medical students whom I supervised, some 400 medical charts from three types of hospitals, (university, city, and Veterans Administration) were assessed, and less than 10% had anything resembling an appropriate work history recorded. As for environmental exposures, other than asking about smoking and alcohol use, virtually nothing could ever be found in the charts on patient hobbies, geographic location of their homes near exposure sources, such as waste disposal sites, or even about second‐hand smoke or other household toxins. Since traditionally physicians are more concerned with treating the conditions they diagnose than determining etiology, for some individuals not recognizing a workplace exposure leading to serious illness might be depriving their patients of potential compensation that would aid them and their families. Unfortunately, even today, many physicians want nothing to do with work‐related illnesses or the potential of having to testify.

This does not mean that this was always the case. In the early 1950s, the United Mine Workers (UMW) bargained successfully for health insurance for coal miners and their families. A few years later, the UMW opened ten new hospitals throughout Appalachia, mostly in Kentucky but with some in West Virginia, and a hospital in Virginia.^101^, ^102^ With the opening of these hospitals, the care of miners and their families changed dramatically. From 60% of births occurring in hospitals, 90% were now found there, and the number of infants dying within the first year of life in Appalachia went from 36 to 9 per 1000 children. Hospital beds overall increased by more than 50% and the number of healthcare workers in the region more than doubled. Unfortunately, as time went on and hospital care became more complicated and expensive, the UMW could no longer sustain the hospitals. The Appalachian Regional Healthcare System took over many hospitals and continues today to provide healthcare in the region, but no longer has its focus and roots devoted to working men and women. Historically, railroads in the United States also ran hospitals for their workers but they too no longer exist.^103^

**Global Perspectives**

It is only in some parts of Europe that there are now hospitals and clinics entirely devoted to the illnesses and diseases of workers and some have existed for considerable periods of time. In Milan there is the Clinica del Lavoro Luigi Devoto that was founded in 1902 as a major health facility devoted to workers' health. In Germany and elsewhere in Europe there are major occupational health clinics at numerous medical institutions. As noted above, in the United States, not all medical schools have even a single physician trained in occupational medicine, and occupational medicine clinics at hospitals are not a major focus of any institution in the United States.

Around the world, the effect of unions and their insurance programs are still important. There are outpatient clinics still operating in the United States, especially in New York State, that primarily look after workers. Unions have also funded and operate clinics connected to industrial work populations. In India, the Employees State Insurance (ESI) program, a scheme devoted to organized workers in India, operates clinics and hospitals around the country that looks after some 55 million workers and family members. In Israel there is a history of socialized occupational medical services.^104^ However, just as in the United States, Canada, India, and most other countries around the world, there is only a small cadre of physicians who are devoted to occupational medicine, many without significant training.

Guidelines for how countries can operate occupational health programs have been promulgated by the World Heath Organization^105^ and in some countries, agencies equivalent to the USA‐based NIOSH or OSHA have been established. In addition to these governmental entities, NGOs with an occupational health remit have arisen. One such NGO that has linked occupational physicians and environmental health scientists around the world was started by Dr. Selikoff together with Dr. Cesare Maltoni. Based in Carpi, Italy, Bernadino Ramazzini's birthplace in 1643, the Collegium Ramazzini counts members from most continents and focuses on promulgating important information on worker health and environmental issues that cover a wide variety of topics, included migrant worker health, the health effects of asbestos, benzene, and other significant exposure hazards. As its first president, Dr. Selikoff set the tone for this organization and its international activities.

This organization stands in contrast to more traditional occupational physicians working for companies who rarely wish to counteract the tendency of companies to hide hazards. These issues have been well documented by Michaels.^106^, ^107^ Insurance companies, like many such entities, are loathe to pay out funds to injured workers, even after court judgments. Perhaps it is because of the principle of “academic freedom” that the most aggressive and socially responsible physicians in occupational medicine can generally be found in academic settings without the constraints of corporate masters or governmental structures, although some “academics” have become allies to industry in obfuscating hazards.^108^ For‐profit companies have also done well in using corporate funds to hide corporate misdeeds.

As a student trained by Dr. Selikoff and as a member of the Collegium Ramazzini, I try to continue bringing insights into settings around the world. I will highlight just a few of these. In India, as noted above, the ESI system employs some 7500 physicians, but up until recently, not a single physician in their system had been trained in occupational medicine. Through collaborative activities with the Maulana Azad Medical College, in New Delhi, the one medical school of about 400 in India that had a trained full‐time occupational physician on staff, I participated over several years of three‐month training courses to certify, with minimal qualifications, physicians who could then claim to be “trained” in occupational medicine. Most of the participants, about 25 per class, were already employed in corporate settings and went through this training for additional certification and then returned to the jobs they had already been doing, but hopefully with a broader base of knowledge and resources.

One physician from the EIS Hospital in Delhi participated in such a course and became trained in the basic principles of occupational medicine and took this training back into the EIS system. Through the joint efforts of the Centre at Maulana Azad and the EIS Hospital, additional training was provided to EIS physicians around the country through short courses, in person and by video conference, to give them a basic understanding of the principles of occupational medicine. Topics covered included history taking, occupational lung diseases, occupational cancers, workplace‐related eye injuries, and similar topics. Just as in America where no requirement exists for training medical students in occupational medicine, in India there is also no such requirement. It should also be noted that many Indian corporations operate major health clinics for their workers and family members, and include many medical specialties, and some even operate their own small hospitals.

Similarly, in China, another aspect of the lack of medical training in occupational medicine became apparent. In many countries public health and preventive medicine has largely been separated from clinical medicine and unless one trained in a public health setting little about exposures in workplaces or the general environment ever entered the medical curriculum, although some such training might be found in separate public health settings. By separating public health and preventive medicine in large part from the rest of clinical medicine in China, the United States, and elsewhere, little if any traditional training in occupational medicine occurred. Interestingly, when I was afforded the opportunity to share information in China, and elsewhere, it was either in a governmental health clinic setting or at a school of public health, not as an integral part of local medical education.

Interestingly, there are two countries that I have visited where governmental agencies were set up to evaluate occupational health issues among workers in those countries. Mongolia and Brunei have established centralized governmental entities where workers are sent from around the country to evaluate potential workplace illnesses and as needed, provided care and compensation for injured workers. When I have lectured in Mongolia it was through the School of Public Health, but I also had the opportunity of interacting with the government health officials and with those physicians intimately involved with these worker evaluations. In Brunei, I also interacted with government officials but did have the opportunity of lecturing to medical students on occupational medicine, and there were several physicians on staff at one of the medical colleges who had been formally trained in occupational medicine.

My experiences in South America have been little different. In Brazil I have lectured to government officials and at scientific meetings, but I am unaware of any occupational medicine curriculum in their medical schools. Similarly, that situation exists at the National Medical University in Bogota, Colombia where no one on staff was formally trained in occupational medicine, and I also found no such faculty member at one of the private medical colleges.

**Conclusions: A Sad State**

While it is obvious that there is now considerable worldwide concern about environmental issues such as climate change, and even some growing concern about injuries and illnesses at workplaces, these matters have not made their way into medical curricula in any significant way. Several solutions can address this issue. First, there needs to be additional worldwide support for the training of occupational physicians. In the United States, a serious effort should be undertaken to once again increase the number of training programs that have shuttered as the budgets for such training from institutions such as NIOSH have diminished over time. Another significant funding change could allow preventive medicine training to be funded, as are all other residents, under Medicare. All other clinical residency programs are supported by the federal government, with the exception of positions in preventive medicine, including occupational medicine.

Also, accrediting bodies for medical schools could require training in occupational medicine as part of every curriculum. Some medical schools do not have independent departments of preventive or community medicine, and these activities may be tied to other departments, such as family medicine or internal medicine. Not only could there be a mandate for such training, but this could be reinforced on the exams that are universally required for the licensure of physicians, with the requirement that such content be included. In countries overseas there are often hierarchical bodies that mandate the components of the medical curriculum, and these organizations could require some occupational training in their medical schools as well. A more overarching collaboration could see that public health, and its occupational and environmental health component, be better integrated into medical education and that the two entities of clinical medicine and public health and preventive medicine are not kept in such separated educational silos.

Lastly, if other entities in the world of work, such as labor unions, become stronger once again, pressure could be put on companies to provide safer and healthier workplaces. OSHA should be able to site‐visit workplaces more than once every 100 years, which is the current situation given present staffing and budget constraints. Around the world the too‐often‐seen concept of “disposable workers” must be replaced by a meaningful valuing of each human life.

Although one would wish that the training for, and provision of, occupational medical services has changed for the better over the past decades, no such developments have taken place.

## 7 THE EVOLVING TOOLMAKER: IN DIALECTICS OF BIOLOGY, HISTORY, AND EHTICS, HUMANKIND SURVIVES BY CHANGING TOOLS OF MIND AND MATTER

**Dedicated to the memory of Irving J. Selikoff, MD**

**Sheldon Samuels, A.B.**

On one of Dr. Selikoff's last days, hospitalized, he asked: “Is Goethe's own last plea for more light an optimism implying possible light at the edge of the eternal abyss of mankind?”

He found an answer in work of Darwin that revolutionized medical research through well‐founded authentication of forms of biological development in populations, concepts proven to be fruitful in the study of disease and distorted social development of afflicted populations. “Darwin's population perspective,” Selikoff would note, unfortunately is often poorly understood. Thus, the expression “survival of the fittest”^109^ is not an expression used by Darwin, the social meaning of which is not consistent with his social views. Yet the expression has become irrationally linked to Darwin in rationalizations of moral reality.

Selikoff's often repeated assertion of the nature of social formation, of population thinking that “all began with Darwin,” incorporated the holistic social—biological integrated perspective of Darwin himself, not the tooth and claw version of the pseudo‐Darwinians.

Darwin, Selikoff noted, found “truth” in the universal struggle for life. The positive checks—war, famine, endemics—raise the mortality rate. Moral restraints, he believed, reduce the rate. He would give the struggle to control asbestos as an example but noted that success in that struggle too often leans on “professionals” in a “think tank” claque who make moral mistakes. Thus, the necessity to challenge myths of professional objectivity, informed by insights such as those of Ludwik Fleck.^109^” The “accumulated experience—not only of an individual but of a well‐trained collective,” Fleck wrote, is one in which the members “teach others to see” ‘facts’.^109^ We need not look far to find examples of the process by which members of a thought collective learn how to “see” facts. Fleck noted the mechanism of teaching “facts”: the incestuous imprinting of thought during the migration of ideas throughout their collectives, driven by the natural striving for the collective rewards of collective achievement.^109^

There is a price to be paid for failure to heed this warning, as seen in efforts to contain the Covid‐19 epidemic by increasing herd immunity. Immunization campaigns, by raising the level of herd immunity, protect not only individuals but entire populations. If the number susceptible is close to zero, there is little likelihood of epidemic spread because there are not enough susceptible in the pool. If a sufficiently high proportion of the population is immune to an infectious disease, the probability that an infectious agent will encounter a susceptible host is reduced. Survival of the organism may no longer be jeopardized. Infection tends to die out.

Unfortunately, behaviors of some can be transmitted like agents of contagious disease. The conscious spread of untruths about methods of preventing disease—such as Covid‐19 immunization—makes the spewing agent an agent of anomie [social chaos] with resulting disease, reducing the advent of herd immunity.

If herd immunity declines, disease and death increase, raising moral issues on supposed rights of free unimpeded speech or expression. More, self‐contagion of the spewing agent increases disease and death among the sub‐population of spewers of untruths. The likelihood of herd immunity is increased by their suicides.

The increase in such social suicide, reflecting social chaos he labeled “anomie,” was described by Durkheim in a series of publications initiated in 1893.^38^ Its long conceptual life has not reduced its importance in understanding this dynamic of human communities.

The rite of cost–benefit analysis is another example of such suicide through the collective abuse of ideas. Practitioners demand precise quantification beyond the capacity of the subject matter to support precision and quantification, by setting arbitrary monetary values for a human life. Thus, there can be no agreement among the regulatory agencies or their overseers among elected officials on the actual value of a life. The practitioners typically ignore guidelines enabling ethical considerations ordered in U.S. Supreme Court decisions. A plurality in the case of a workplace standard for benzene exposure made clear that the calculation of significant risk [to set priorities in standard‐setting] need not be “a mathematical straitjacket,” but can be “based on policy considerations… risking error on the side of overprotection rather than under‐protection.”^110^ Court decisions have not stopped the use of these analyses. The irrationality of cost/benefit analysis is cogently summarized by a leading moral philosopher—the late Alan Gewirth—in one of his final papers.^111^ Cost/benefit analysis assumes that all the variables with which it deals are or can be made commensurable with one another so that there is a common denominator into which the costs and benefits can be translated: a person's preferences in the allocation of resources, for example, in allocation of money. He found five difficulties with this position:

- Distributive justice.
- Knowledge.
- No market price.
- Those who set a price on human life are not those being valued.
- Permits polluters to pay to prolong the risk.

Social suicide through desecration of reason embodied in cost–benefit analysis prevails not only in United States regulatory policy but also in setting priorities in the nation's budget. This is done by accepting a skewed meaning of “growth,” whether in sociological, biological, or economic dialogue.

Issues of science and ethics that beset society have at their core ideas central to the dialectic. Combining natural and moral philosophy in research and teachings on the progressive character of the descent of man yields the idea that man is naturally supplied with a special moral sense, a Darwinian factor in the evolution of the civilized human, demonstrating that moral phenomena are no less real than their physical expression. Our chronic failure to prevent disease associated with the sea of toxic agents in the community and work environments in great measure can be attributed to our failure to link natural with moral phenomena which in a critical sense are no less ‘natural’.

The pleasures of health, freedom from pain, self‐expression, years of existence, and reverence for our families, neighbors, and fellow workers and other humans we have never met, and cannot name, embody something called “life.” The growth or increase in mere quantity of these elements of our well‐being in progressive development are no less real in linked populations than the differential development of organs and infrastructure [epigenesis] in the individual. The mature individual, like the mature society is thus enabled to support the complex needs of the whole. The chance for life for some of us, and all of us, is thus embodied in the chances for life of one of us.

**Moral Instinct**

November 1, 2012, in a pilgrimage to Darwin's crypt in London's Westminster Abbey, this writer found aisles flooded with hundreds of visitors to this great and beautiful house of worship. For many, it would not be a place for remembering Darwin. The primary attack on his findings of human evolution was led by Bishop Wilberforce, a Dean of Westminster. Ironically, Darwin had spent 3 years at Christ's College, Cambridge studying to qualify for the Anglican ministry before leaving on the voyage of the Beagle that took him into humanities past and future. He expected a career in a parish, and he was qualified for that career. He did not expect that the last years of his life would be spent dissecting thousands of barnacles, seeking clues to the origin of species of creature life.

What he thought would be a youthful break at sea became a departure from a church whose members believed in the genesis and the flood of the Tanach, the Old Testament, literally as written. Orthodox leaders of religious faith condemned his refutation of the account of human creation in Genesis. Darwin never attended the great debates in which he was unjustly ridiculed. He knew what was to come. When he died, as he wished, they were to have buried him near his home in Down, in an ancient church yard. Twenty members of the House of Commons thought differently. They demanded the same treatment England's other scientific leaders received: a crypt in the Abbey of Westminster. The church resisted, but the separation of church and state was narrow, and so he was given a well‐attended burial in the floor of the Abbey.

There are virtual monuments to the other great scientists buried there, easily found. We asked directions three times before finding, a few feet from a large memorial of Newton, a small plain white sheet of floor stone, simply chiseled with only his name and dates of birth and death:

Charles Robert Darwin

Born 12 February 1809

Died 19 April 1882

I spotted a priest and asked why the different treatment of Newton and Darwin. Newton was at least as revolutionary. He verified the finding that Earth [and thus Man] is not the center of the universe, plus a system of causation that excludes special divine interventions, both contrary to orthodox belief but consistent with Darwinian belief.

The priest said: “Well, you know that he was buried amid much controversy.” “But, I countered, “that was more than a century ago.” The answer: “He remains buried amid much controversy.”

I said no more but thought that we must mourn more than the dead. We must mourn the living absurdities of our species, absurdities that take life itself, the preservation of which ought to be our civilization's highest value, as taught by all the great leaders of western religion.

I returned to the crypt in the floor and looked down again, this time the sounds of the multitude around me drowned out, leaving me to concentrate my thoughts. But what should I think? I sought words of the prayer for the dead. None came to me, but the meditation deepened. I glanced up to the great vaulting windows and around, stopping at beams of light‐filled with something, a crystal of thought: the similarity of Goethe's and Selikoff's pleas for “more light.” Selikoff's plea was Darwin's “more light.” But the nights seem simply to grow longer.

Ought we seek more light? Where? Rays might find their way through new crystals of thought, through windows of mind in the vaulted ceiling of the Temple of Truth. In the Age of the Atom, must the nuclear swords of darkness reap death, or could buds of life open in a sun not yet darkened?

The nurturing of a Darwin was in the hands of many artisans of great minds and teaching institutions. In this case, the University of Edinburgh, where Darwin and his father and grandfather studied medicine and natural philosophy. Together, they absorbed empirical developmental biology in an evolving conception of microcosmic and macrocosmic developmental systems in natural history and human evolution. The theoretical constructs of Darwin's population thinking found its way to another student at Edinburgh's medical school only three generations later: Irving J. Selikoff.

Historically, the issues and the tools of resolution are unconcealed in our evolving human consciousness. They manifest in ancient disputes such as the meaning of developmental change, as in epigenesis versus preformed growth among biologists. They take place in *ecumenes*: areas marked by inter‐communicating populations. In an ecumene centered in Elea, near Naples, 25 centuries ago, *Parmenides* sought the good and beautiful Goddess of Truth in the Temple of Nature. There the axiomatic systems of science and philosophy were examined. Then and now, answers to the questions found in hypotheses of the scientist and assumptions of philosophers are judged not only by their fruitfulness, but by their rejection of chaos as the dominant character of the universe. Traditions within an ecumene themselves evolve, as our ability to find answers change over time in response to the questions asked, some of which do not change.

Correcting mistakes that plague application of Darwin's moral and scientific thinking in the reduction of environmental risk had long been a concern but became a preoccupation in Selikoff's final months of life. As a physician, his days had been spent meeting the needs of his patients, who more often than not were also his research subjects. As a scientist, he had struggled to promote acceptance of multifactor‐deterministic causation. As a human, he sought exorcism of a death‐perpetuating ghost in the thought collectives of our professions seen on the moral surface of human consciousness.

“Is the result of our work in environmental health,” asked Selikoff, “to end as mere entries of economic price balanced in the ledgers of industry and government?” Selikoff's answer was optimistic.

We can correct this distortion of our work, he believed, a distortion of what Darwin's population thinking means found in the belief that variations naturally selected are like petals of cherry blossoms wafting in directionless winds of spontaneity, falling we cannot know where, a frequent mistake with more than methodological implications. The social effects of distorting the role of chance and necessity in multi‐factor causation led to the accounts of tooth‐and‐claw behaviors of pseudo‐Darwinism. They slow mankind's evolving moral sense. They mock Darwin and those of us who are lifted in awe of his memory to see the very essence of evolution: the struggle of and for life itself guided by moral instinct.

The moral instinct is drawn from the aggregation called “society,” the structure analyzed by Aristotle (born 384 BC), in which the basic unit is the family.

*“The family is the association established by nature for the supply of men's every day wants. … But when several families are united, and the association aims at more than the supply of daily needs, the first society to be formed is the village. And the most natural form of the village appears to be that of a colony from the family … When several villages are united in a single complete community … the state comes into existence … continuing in existence for the sake of a good life*.

*… And it is a characteristic of man that he alone has any sense of good and evil, of just and unjust … and the association of living beings who have this sense makes a family and a state.”* ^18^

**Anomie.**

Despite broadening recognition of Darwin's personal and family history of human rights advocacy, mistakes about his work continue to draw blood. The wide‐spread use of the term “Social Darwinism” is common, a symptom of the anomie identified by Durkheim.^38^ Normally the division of labor, he noted, produces social solidarity. Sometimes it presents pathological forms that negate solidarity, such as disunity in the ideas and methods of science. Unity is an indispensable condition of happiness built on spontaneous consensus. “But it appears certain that happiness is something besides a sum of pleasure. … All pleasure is a sort of crisis … Life, on the contrary, is continuous. What happiness expresses is … the health of physical and moral life in its entirety.” “… [W]hat ever may be part of hope in the genesis of the instinct of conservation,” he wrote, “is a piercing witness of the relative bounty of life. [Yet when] evil increases or the causes of suffering increase, or the relative force of individuals is reduced, then as this sentiment passes in societies … with one stroke we shall be able] to measure those of the average unhappiness in these same environments. This fact is the number of suicides.” …

“But suicide scarcely appears except with civilization.” … “It is not an act of despair but of abnegation. … In all these circumstances, man kills himself, not because he judges life is bad, but because the ideal … demands the sacrifice.” Total harmony is found in the coercive force of ethical conduct. “Law and morality are the totality of ties which bind us to society.”^38^

The message of anomie, carried by ancient media like the Eleatic dialectic, enters *ecumenes* marked by inter‐communicating populations embedded with thought collectives who examine the axiomatic systems of science and philosophy. Answers to the questions found in hypotheses of the scientist and assumptions of philosophers are judged not only by their fruitfulness, but by their rejection of chaos as the dominant character of the universe. Then and now, traditions within an ecumene themselves evolve, as our ability to find answers change over time in response to the questions asked, some of which do not change.

Over centuries and continents, the Eleatic ecumene remains remarkably intact. Witness Einstein's account of the discovery of the theory of relativity:

“Today everyone knows, of course, that all attempts to clarify this paradox [of light that leads to special relativity] satisfactorily were condemned to failure as long as the axiom of the absolute character of time, or of simultaneity, was rooted unrecognized in the unconscious. To recognize clearly this axiom and its arbitrary character already implies the essentials of the solution of the problem.”^22^

To this history we must add the caution of Leo Strauss: “If principles are sufficiently justified by the fact that they are accepted by a society, the principles of cannibalism are as defensible or sound as those of civilized life.”^112^

**The Genesis of Hope**

In *Philosophie des Als Ob*, Vaihinger argued that human beings can never really know the underlying reality of the world, and that as a result we construct systems of thought and then assume that these match reality. We behave "as if" the world matches our models.^15^ In particular, he used examples from the physical sciences, such as protons, electrons, and electromagnetic waves. None of these phenomena have been observed directly, but science pretends that they exist, and uses observations made on these assumptions to create new and better constructs. The heuristic fictions of Kant's theory of method in his *Critique of Pure Reason* find full expression a century later in the work of Vaihinger. The fictional nature of general ideas: “the whole world of ideas is an instrument to enable us to orientate ourselves in the real world, but is a copy of that world … [they are] relatively objective ideational constructs [not] subjective or fictional ….”^15^

The difference between poetic fiction and a scientist's hypothesis is critical, for example, when Darwinian hypothesis is verified fiction disappears, resulting in real explanation. Poetry is eternal. Goethe's schematic animal archetype is a fiction justified as an expedient, according to Vaihinger, albeit Goethe himself saw it differently: dual possibilities within both the universes of “as if” and “as is.”

“In doing this, I soon felt the necessity of establishing a type,” the poet‐scientist wrote from the perspective of “as is,” in describing his search for real primordial starting points, “against which one might gauge all mammals for conformity and deviation; and just as I had once sought out the archetypal plant, I now sought to find the archetypal animal.” But in his search he understood that if his findings were to be systematized and explained, not just an empirical, but a conceptual dimension is necessary.

Thus, Goethe claimed, “in an attempt to study the laws whereby life is given to organic nature … quite justifiably, a force was ascribed to this life for purposes of discourse; and this force could be, indeed had to be, assumed. We [are] obliged to assume a double point of view, considering ourselves as an entity sometimes perceivable by the senses, and at other times recognizable only with the inner sense or noticed only by an effect.”^16^

Causation as a law of nature is an eternal idea the variations of which bridge the worlds of “as if” and “as is.” Between these worlds is an abyss‐never‐filled, nor yet a void, expanding with the expansion of technology over the long eons of human evolution with mounting catastrophic strife in the struggle for life and freedom. Clearly illustrated in the history of controlling atomic technologies, now centuries long, our failures multiply at an ever‐quickening pace from the difficulties of controlling our hands and their extensions—simple tools and complex technologies—with mind and reason. The living human being is not a preformed machine built, boxed, and controlled by an isolated will for use at random or self‐selected time. Each and together we are organic hierarchies of formative processes. Formation is not explained by function, which explains usefulness. Use is not its cause or a mechanism, but a description of survival value for the individual and the population bearing the useful trait.

## DISCLOSURE BY AJIM EDITOR OF RECORD

John Meyer declares that he has no conflict of interest in the review and publication decision regarding this article.

## AUTHOR CONTRIBUTIONS

All four authors reviewed and approved the final manuscript. All four authors contributed to the Introduction. Sheldon Samuels wrote the following: Conversations with Irving's Ghost: The Struggle for More Light ‐ Dialogues with Dr. Selikoff Reconstructed. History of the Atomic Weapons Workers' Fight for Compensation: A Report from the Abyss of Occupational and Environmental Health. And The Evolving Toolmaker: In dialectics of biology, history and ethics, humankind survives by changing tools of mind and matter. Dedicated to the memory of Irving J. Selikoff, MD. William N. Rom wrote the Abstract and Global Warming: An Opportunity for Unions and Government to Embrace Renewable Energy Jobs. Knut Ringen wrote: The Future of Nuclear Power. Arthur Frank wrote: Worldwide Changes in Occupational Health Provision Over the Past Half Century. All four authors thank the reviewers and the editor, John Meyer, for helpful suggestions and editing.

## Data Availability

The data that support the findings of this study are openly available in public data at https://www.apple.com/.
